# Fungal Unspecific Peroxygenases Oxidize the Majority of Organic EPA Priority Pollutants

**DOI:** 10.3389/fmicb.2017.01463

**Published:** 2017-08-09

**Authors:** Alexander Karich, René Ullrich, Katrin Scheibner, Martin Hofrichter

**Affiliations:** ^1^Department of Bio-and Environmental Sciences, Technische Universität Dresden–International Institute Zittau Zittau, Germany; ^2^Enzyme Technology Unit, Brandenburg University of Technology Cottbus, Germany

**Keywords:** EC 1.11.2.1, peroxidase, xenobiotics, chlorobenzene, nitroaromatics, polycyclic aromatic hydrocarbons, fungi

## Abstract

Unspecific peroxygenases (UPOs) are secreted fungal enzymes with promiscuity for oxygen transfer and oxidation reactions. Functionally, they represent hybrids of P450 monooxygenases and heme peroxidases; phylogenetically they belong to the family of heme-thiolate peroxidases. Two UPOs from the basidiomycetous fungi *Agrocybe aegerita* (*Aae*UPO) and *Marasmius rotula* (*Mro*UPO) converted 35 out of 40 compounds listed as EPA priority pollutants, including chlorinated benzenes and their derivatives, halogenated biphenyl ethers, nitroaromatic compounds, polycyclic aromatic hydrocarbons (PAHs) and phthalic acid derivatives. These oxygenations and oxidations resulted in diverse products and—if at all—were limited for three reasons: (i) steric hindrance caused by multiple substitutions or bulkiness of the compound as such (e.g., hexachlorobenzene or large PAHs), (ii) strong inactivation of aromatic rings (e.g., nitrobenzene), and (iii) low water solubility (e.g., complex arenes). The general outcome of our study is that UPOs can be considered as extracellular counterparts of intracellular monooxygenases, both with respect to catalyzed reactions and catalytic versatility. Therefore, they should be taken into consideration as a relevant biocatalytic detoxification and biodegradation tool used by fungi when confronted with toxins, xenobiotics and pollutants in their natural environments.

## Introduction

The most important classes of organic pollutants in the environment are mineral oil constituents as well as halogenated and nitrated products of petrochemicals. Enzymatic transformation and degradation of such recalcitrant compounds, many of them xenobiotics *in sensu stricto*, generally proceeds via two modes: reductive and oxidative attack (Spain, 1995; Durán and Esposito, 2000; Ye et al., 2004). Oxidoreductases, (e.g., dehydrogenases and oxygenases/[per]oxidases, respectively) play key roles in both degradative strategies and have been well studied (Durán and Esposito, 2000). Oxidases, oxygenases and peroxidases (POX) can be classified according to their co-substrates; the most important of them—involved in the aerobic degradation of numerous organic pollutants—are shortly discussed below.

Polyphenol oxidases, i.e., laccase (LAC) and tyrosinase (TYR), are copper containing enzymes that catalyze the oxidation of phenolic compounds with dioxygen (O_2_) as electron acceptor and without the need of additional co-enzymes, such as NAD(P)H. They are found in almost all domains of aerobic life and fulfill diverse metabolic functions (Ullrich and Hofrichter, 2007). Whereas, LAC does not directly incorporate oxygen into substrates, TYR can do so along with phenol/catechol oxidation (Jergil et al., 1983; Majcherczyk et al., 1998). Nevertheless, also LAC may indirectly oxyfunctionalize molecules, for example, polycyclic aromatic hydrocarbons (PAHs) or phenols, via one-electron oxidation followed by disproportionation and water addition (Majcherczyk et al., 1998; Wu et al., 2008).

Oxidases using O_2_ and reduced co-enzymes (e.g., NAD(P)H or FADH_2_) as oxygen donor (electron acceptor) and electron donor, respectively, are usually referred to as oxygenases (Guengerich, 2001). In dependence on the number of oxygen atoms introduced into the substrate molecule, monooxygenases, and dioxygenases are distinguished. Cytochrome-P450 monooxygenases (P450s) are heme-thiolate proteins where a porphyrin moiety (iron protoporphorin IX; heme) is ligated via a cysteine residue to the polypeptide chain (α-helix) of the apo-enzyme (Munro et al., 2012). The protein superfamily of P450s is highly diverse and comprises versatile intracellular biocatalysts found in all domains of life (Anzenbacher and Anzenbacherová, 2001; Meunier et al., 2004; Munro et al., 2012), and even virally encoded P450s have been described (Lamb et al., 2009). P450s usually utilize NAD(P)H as electron-delivering co-substrate but some of them can also catalyze monooxygenations with peroxides as oxygen donor via the so-called shunt pathway (Munro et al., 2012). While some P450s play specific anabolic roles, e.g., in sterol biosynthesis (Lepesheva and Waterman, 2004), others are rather unspecific and involved in the metabolism of xenobiotics, toxins and drugs (Anzenbacher and Anzenbacherová, 2001; Guengerich, 2001).

Multicomponent monooxygenases (BMMs) represent another family of versatile biocatalysts transferring oxygen to various substrates (Leahy et al., 2003). Thus, toluene 4-monooxygenase and methane monooxygenase are capable of oxygenating—in addition to their eponymous substrates—diverse alkenes and arenes including hardly reactive benzene (Whited and Gibson, 1991; Sazinsky et al., 2004). BMMs have only been found (and characterized) in bacteria and archaea so far (Notomista et al., 2003). Other non-heme monooxygenases contain flavin as prosthetic group (FMOs) (van Berkel et al., 2006; Huijbers et al., 2014). They activate O_2_ with a reduced flavin cofactor to form a peroxyflavin that attacks the substrate (van Berkel et al., 2006). As P450s, FMOs are found in bacteria and eukaryotes (van Berkel et al., 2006).

Oxygenases that catalyze the incorporation of the entire O_2_ molecule are called dioxygenases (DIOXs). Most DIOXs are iron containing enzymes, e.g., Rieske-type DIOXs (also referred to as arene DIOXs) (Bugg and Ramaswamy, 2008). Rieske-type DIOXs contain a [2Fe-2S] cluster and preferably catalyze the formation of *cis*-dihydroxylated metabolites (Ferraro et al., 2005). Arene DIOXs are capable of oxidizing inactivated arenes, such as toluene, benzene and even nitrobenzene (Lessner et al., 2002; Bagnéris et al., 2005).

All types of oxygenases can be involved in the detoxification and biodegradation of organic pollutants and xenobiotics by microorganisms and often they initiate catabolic pathways resulting in the utilization of these compounds as soles carbon and energy sources (Fewson, 1988; Copley, 2000). In this context, the incorporation of oxygen does not only activate the molecules but also increases the compounds' water solubility and hence their bioavailability. That way, many compounds listed as EPA priority pollutants, such as benzene and its derivatives, become accessible to enzymatic attack by other enzymes (e.g., ring-fission enzymes, POX or LAC) upon hydroxylation.

Secreted peroxidases, such as fungal lignin peroxidase (LIP), manganese peroxidase (MNP), and versatile peroxidase (VP), plant horseradish peroxidase (HRP), animal dehaloperoxidase and lactoperoxidase as well as bacterial and fungal dye-decolorizing peroxidases (DYPs), are typical degradative and detoxifying biocatalysts that utilize hydrogen peroxide as electron acceptor (Camarero et al., 1999; Piontek et al., 2001; Hofrichter, 2002; Osborne et al., 2009; Strittmatter et al., 2011). They all contain heme as prosthetic group that is linked via a proximal histidine to the polypeptide chain (heme-imidazole POX) (Ullrich and Hofrichter, 2007). Heme peroxidases are known from all kingdoms of life (Vlasits et al., 2010). Because of the involvement of some of them in lignin biodegradation (MNP, LIP, and VP), which opens an eco-physiological niche for specialized fungi (basidiomycetous white-rotters and litter-decomposers) and is of general interest for the pulp and paper sector, they have been intensely studied over the last three decades (Kirk and Farrell, 1987; Hofrichter, 2002; Martínez et al., 2009). Interestingly, it has recently been proposed that the appearance of fungal ligninolytic peroxidases led to the end of the carboniferous period (Floudas et al., 2012). In addition to lignin, these enzymes were found to efficiently oxidize diverse organic pollutants as well and hence were proposed to be part of unspecific bioremediation/bioattenuation systems in nature (Pointing, 2001; Hammel and Cullen, 2008; Qayyum et al., 2009; Harms et al., 2011).

Unlike the ligninolytic heme peroxidases, the heme iron of chloroperoxidase (CPO) from the ascomycete *Caldariomyces (Leptoxyphium) fumago* (Dawson, 1988) is linked to a cysteine (heme-thiolate peroxidase-HTP), as in the case of P450s. In 2004, a new HTP type was discovered, which is presently known as unspecific peroxygenase (UPO) (Ullrich et al., 2004; Ullrich and Hofrichter, 2005; Hofrichter and Ullrich, 2006) representing a functional hybrid of peroxidases and P450s (Hofrichter and Ullrich, 2006; Hofrichter et al., 2015). Besides prototypical peroxidase reactions like one electron oxidations, UPO transfers hydrogen peroxide-borne oxygen and catalyze various hydroxylations, e.g., of aromatic and aliphatic hydrocarbons (Kluge et al., 2009, 2012; Aranda et al., 2010; Kinne et al., 2010; Peter et al., 2011; Karich et al., 2013). Moreover, epoxidation, sulfoxidation, heterocyclic *N*-oxidation, and ether cleavage (*O*-dealkylation) have been reported (Ullrich et al., 2008; Aranda et al., 2009; Kinne et al., 2009b; Kluge et al., 2012), and moreover, UPO has catalase and haloperoxidase activities (Hofrichter et al., 2015). Thus, UPO combines features of LAC/POX (one-electron oxidation), monooxygenases (incorporation of one oxygen atom into the substrate) and POX/catalase (H_2_O_2_ as co-substrate) and hence represents a multifunctional type of oxidoreductase with almost catalytic promiscuity (Pandya et al., 2014; Hofrichter et al., 2015).

UPO genes are ubiquitous in the fungal kingdom (Eumycota) and beyond that, have only been found in the (super) phyllum of heteroconta (e.g., in fungus-like Peronosporales belonging to the former class of oomycetes and in a few diatoms) (Pecyna, 2016). Horizontal gene transfer from ascomycetes was proposed to be the most probable explanation for the occurrence of UPO genes in the latter organisms, leading to the conclusion that they are an autapomorphic feature in the kingdom of fungi (Pecyna, 2016).

**Figure 1:**
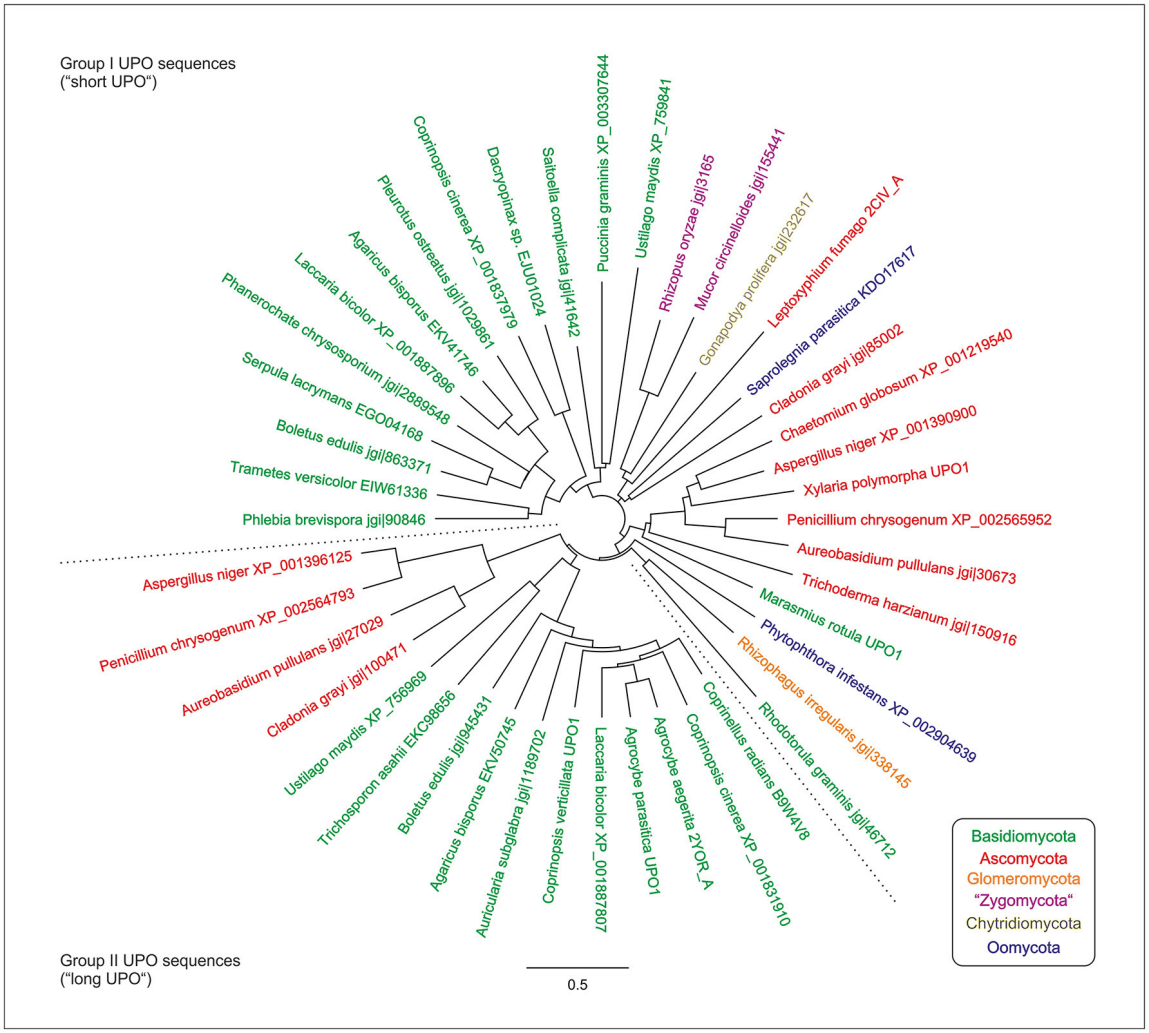
Neighbor-joining phylogenetic tree of UPO/HTP-sequences using Jukes-Cantor genetic distances; updated according to Hofrichter et al. (2015). Green, Basidiomycota; red, Ascomycota; blue, “Oomycota” (Peronosporales, Saprolegiales); purple, “Zygomycota” (Mucoromycotina); dark blue, Chytridiomycota; and orange, Glomeromycota. The dotted lines separate UPO sequences of groups I and II (short and long UPOs, respectively).

Phylogenetically, Unspecific peroxygenases (UPOs) can be classified into two large groups/families, which differ, among others, in molecular size: (i) group I, the short UPOs with an average mass of 29 kDa and (ii) group II, the long UPOs with an average mass of 44 kDa (compare Figure 1; Hofrichter et al., 2015). The latter are exclusively found in ascomycetes and basidiomycetes, while the former are distributed among all fungal phyla (compare Figure 1; Hofrichter et al., 2015). The herein studied UPOs of *Agrocybe aegerita* (*Aae*UPO) and *Marasmius rotula* (*Mro*UPO) belong to the long and short basidiomycetous UPOs, respectively. Interestingly, the above-mentioned CPO, which had been an “orphan” among the heme peroxidases for decades, can now be classified into group I of UPOs/HTPs as well.

Against the background of widespread occurrence of UPOs in the fungal kingdom and their catalytic versatility, it is worth to study the conversion of a representative number of organic pollutants by these enzymes. So we have tested here 44 substrates, of which 40 are listed as EPA priority pollutants including chlorinated benzenes, halogenated biphenyl ethers, nitroaromatics, PAHs and phthalates (USEPA, 1979).

## Materials and methods

### Enzyme preparation and chemicals

*Aae*UPO and *Mro*UPO were prepared as described by Ullrich et al. (2004) and Gröbe et al. (2011), respectively. Final enzyme preparations had specific activities of 98 and 28 U/mg for *Aae*UPO and *Mro*UPO, respectively. Chemicals used were purchased from Sigma Aldrich-Germany (Munich, Germany) with the highest purity available.

### Enzyme assay and reaction setup

Enzymatic activity of UPOs was routinely assayed by following the oxidation of veratryl alcohol to veratraldehyde at 310 nm (ε_310_ = 9,300 M^−1^ cm^−1^) in a buffered reaction mixture (pH 7.0) according to Ullrich et al. (2004). Photometric measurements were performed using a Cary Bio 50 spectrophotometer (Varian Inc., Walnut Creek, CA, USA).

Enzymatic reactions were performed in triplicate in 1.5-mL HPLC vials containing 50 mM potassium phosphate buffer (pH 7) and acetronitrile at concentrations between 5 and 30% vol/vol. The substrate concentration ranged from 0.1 to 1 mM, e.g., in the case of large PAHs and phenolic compounds, respectively. The total reaction volume varied between 0.5 and 1 mL. Reactions were started by addition of H_2_O_2_ (final concentration 0.5–2 mM). H_2_O_2_ was added via syringe pumps over 30 min or, in the case of large PAHs, over 2 h. In additional reaction setups, ascorbic acid (4 mM) was added to the reaction mixtures to prevent polymerization starting from intermediate phenoxy radicals formed by one-electron oxidation (Kinne et al., 2009a). The final enzyme activity (*AaeU*PO and *Mro*UPO) in the reaction mixtures ranged between 0.5 and 1 U/mL (veratryl alcohol units measured at pH 7). Controls for each reaction setup were run without enzyme. Detailed information on the reaction setups, analytical methods and some specific results (e.g., HPLC elution profiles) are given in the Supplementary section.

### Sample preparation

Samples from the reaction mixtures were collected 30 min after the reaction had started and stopped by injection into an HPLC system or by addition of 1 mM sodium azide. To ensure dissolving, in a few cases, the reaction mixtures were diluted with acetonitrile (e.g., for the analysis of phthalic acid derivatives or small PAHs, such as acenaphthene) or acetone (e.g., for large PAHs, such as benzo[a]pyrene or benz[a]anthracene) prior to injection.

### HPLC analyses

Reaction products were analyzed by HPLC with MS and/or UV-Vis detection using an Agilent Series 1,200 instrument equipped with a diode array detector (Agilent Technologies Deutschland GmbH, Böblingen, Germany). The HPLC system was generally equipped with reversed phase columns; i.e., Luna C18(2), Synergi polar, Kinetex C18 and Kinetex PFP (each supplied by Phenomenex, Aschaffenburg, Germany). An electrospray ionization mass spectrometer (6310 IonTrap, Agilent Technologies Germany GmbH), in negative and positive mode, was used to determine mass-to-charge ratios of substrates tested and metabolites formed in the course of UPO reactions.

Oxidation products were identified by comparing their retention times, mass and spectral data with authentic standards (so far available) and with literature data. In the absence of standards or reference data, metabolites were tentatively assigned according to their mass and UV-Vis spectra.

## Results and discussion

The main outcome of the enzymatic oxidation tests with two fungal UPOs and diverse EPA organopollutants is summarized in Table 1. This includes the respective type of substrate functionalization (incorporation of oxygen, release of functional groups, one-electron oxidation, etc.), the relative conversion of the tested compounds in semi-quantitative form (five levels of conversion) and the products formed. Polymerization products were observed in all cases, in which phenolic groups were present in the substrates or emerged in the course of the reaction as intermediates (with the exception of 2,4-dinitrophenol).

**Table 1:** EPA priority pollutants (and a few other recalcitrant compounds) tested with respect to their conversion by UPOs, including references to literature data of other oxidoreductases.

**Compound**	Functional groups introduced and/or released^*^	**Relative conversion**		***Oxidases***	**References**
		***Aae*UPO**	***Mro*UPO**		
Chlorobenzene	−OH (1x, 2x)	+	nt	P450; DIOx	de Bont et al., 1986; Nedelcheva et al., 1998
2-Chlorophenol	−OH (1x, 2x)	+++	nt	LAC; HRP; CPO; LIP; MNP; TYR; VP	Wada et al., 1995; Durán and Esposito, 2000; Zhang et al., 2008; Hibi et al., 2012
1,2-Dichlorobenzene	−OH	+	nt	P450; DIOx	Nedelcheva et al., 1998; Jones et al., 2001; Monferran et al., 2007
1,3-Dichlorobenzene	−OH; −Cl	++	nt	P450; DIOx	de Bont et al., 1986; Jones et al., 2001
1,4-Dichlorobenzene	−OH	+	nt	P450; DIOx	de Bont et al., 1986; Spiess et al., 1995; Nedelcheva et al., 1998; Jones et al., 2001; Monferran et al., 2007
2,4-Dichlorophenol	−OH	+	+++	LAC; HRP; LIP; DYP; VP; MNP; P450 FMO	Beadle and Smith, 1982; Valli and Gold, 1991; Schomburg and Stephan, 1994; Yee and Wood, 1997; Xu and Bhandari, 2003; Zhang et al., 2008; Fodil et al., 2011; Hibi et al., 2012
1,2,4-Trichlorobenzene	−OH	++	nt	P450; DIOx	van der Meer et al., 1991; Marco-Urrea et al., 2009
2,4,6-Trichlorophenol	−OH; −Cl	+++	nt	LAC; VP; LIP; MNP; DyP; FMO	Wieser et al., 1997; Reddy et al., 1998; Leontievsky et al., 2001; Fodil et al., 2011; Hibi et al., 2012
Pentachlorophenol	–	0	0	P450; LAC; LIP; MNP; TYR; DYP; VP; FMO	Reddy and Gold, 2000; Thakur et al., 2002; Montiel et al., 2004; Davila-Vazquez et al., 2005; Crawford et al., 2007; Jeon et al., 2008; Fodil et al., 2011
Hexachlorobenzene	–	0	0	P450	Jones et al., 2001
*para-*Chloro-*meta*-cresol	−OH; −Cl	+++	nt	LAC; TYR	Bollag et al., 1988; Freire et al., 2003
2-Chloronaphthalene	−OH (1x, 2x)	+++	nt	P450	Mori et al., 2003
3,3-dichlorobenzidine	−OH	++	nt	P450; FMO	Iba and Thomas, 1988; Imaoka et al., 1997
4-Chlorophenyl phenyl ether	−OH (1x, 2x)	++	nt	P450	Hundt et al., 1999; Hiratsuka et al., 2001
4-Bromophenyl phenyl ether	−OH (1x, 2x)	++	nt	P450	Hundt et al., 1999
3-Chlorophenol^**^	−OH (1x, 2x)	+++	nt	LAC; HRP; CPO; LIP; MNP; TYR; VP	Wada et al., 1995; Durán and Esposito, 2000; Hibi et al., 2012
4-Chlorophenol^**^	−OH (1x, 2x); −Cl	+++	nt	LAC; HRP; CPO; LIP; MNP; TYR; DYP; VP	Wada et al., 1995; Durán and Esposito, 2000; Freire et al., 2003; Zhang et al., 2008; Fodil et al., 2011; Hibi et al., 2012; Liers et al., 2014
Nitrobenzene	–	0	nt	BMM; DIOx	Spain, 1995; Lessner et al., 2002; Fishman et al., 2004; Ye et al., 2004
2-Nitrophenol	−OH	+	nt	BMM; FMO; DYP	Ye et al., 2004; Vardar and Wood, 2005; Xiao et al., 2007; Büttner et al., 2015
4-Nitrophenol	−OH	++	nt	BMM; FMO; P450; DYP	Spain, 1995; Amato et al., 1998; Kadiyala and Spain, 1998; Fishman et al., 2004; Ye et al., 2004; Büttner et al., 2015
2,4-Dinitrophenol	–	0	nt	FMO	Cassidy et al., 1999
2,4-Dinitrotoluene	=O	t	nt	DIOx	Spain, 1995; Johnson et al., 2002; Ye et al., 2004
2,6-Dinitrotoluene	–	t	nt	DIOx	Nishino et al., 2000; Ye et al., 2004
4,6-Dinitro-*o*-cresol	−OH	t	nt	FMO	Cassidy et al., 1999
Benzidine	−OH	t	+	P450; CPO; HRP; LPO; LAC	Phillips and Leonard, 1976; Yamazoe et al., 1988; Lakshmi et al., 1996
1,2-Diphenylhydrazine	−OH	++	nt		
*bis*(2-Ethylhexyl) phthalate	–	0	0	P450	Wittassek and Angerer, 2008
Butyl benzyl phthalate	−OH; = O	+	t		
Di-*n*-butyl phthalate	−OH; = O	+	+	DIOX	Eaton and Ribbons, 1982
Di-*n*-octyl phthalate	−OH; = O	t	t		
Diethyl phthalate	–	0	0		
Dimethyl phthalate	–	0	0		
Acenaphthylene	−OH (1x, 2x); = O	++	++	LAC; DIOx; P450	Majcherczyk et al., 1998; Pinyakong et al., 2004; Shimada et al., 2015
Acenaphthene	−OH (1x, 2x); = O	++	++	LIP; LAC; DIOx; P450	Vazquez-Duhalt et al., 1994; Majcherczyk et al., 1998; Pinyakong et al., 2004; Shimada et al., 2015
Benzo[a]pyrene	−OH (1x, 2x)	+	++	MNP; LAC; LIP; DIOx; P450	Haemmerli et al., 1986; Warshawsky et al., 1988; Bogan and Lamar, 1995; Sack et al., 1997; Kim et al., 1998; Dodor et al., 2004
Benzo[a]anthracene	−OH (1x, 2x)	++	+	MNP; LAC; LIP; P450	Wood et al., 1976; Bogan and Lamar, 1995; Sack et al., 1997; Majcherczyk et al., 1998
Indeno[1,2,3-cd]pyrene	−OH (1x, 2x)	t	t	LAC	Wu et al., 2008
Benzo[b]fluoranthene	−OH	0	t	LAC; LIP	Bogan and Lamar, 1995; Majcherczyk et al., 1998
Benzo[k]fluoranthene	−OH; (1x, 2x)	t	t	LAC; LIP	Bogan and Lamar, 1995; Majcherczyk et al., 1998
Dibenz[a,h]anthracene		0	0	LAC	Wu et al., 2008
Benzo[g,h,i]perylene		0	0	LAC; LIP	Bogan and Lamar, 1995; Wu et al., 2008
Perylene^**^	−OH; (1x, 2x)	t	+	LAC	Majcherczyk et al., 1998
9,10-Dihydrophenanthrene^**^	−OH; (1x, 2x)	t	+		
2,4-Dimethylphenol	−OH; = O	+++	nt	FMO; LAC; HRP	Klibanov et al., 1980; Arenghi et al., 2001; Ghosh et al., 2008
Benzene		+		UPO	Karich et al., 2013
Naphthalene		++		UPO	Kluge et al., 2009
Phenol		+++		UPO	Karich et al., 2013
Anisole		+++		UPO	Kinne, 2010
Toluene		+++		UPO	Kinne et al., 2010
Ethylbenzene		+++		UPO	Kluge et al., 2012
Anthracene				UPO	Aranda et al., 2010
Fluorene		+		UPO	Aranda et al., 2010
Phenanthrene		+		UPO	Aranda et al., 2010
Pyrene		+		UPO	Aranda et al., 2010

Evaluation of relative conversion was done according to the following pattern: no conversion (“0”), trace amounts of products detected (“t”), little conversion—well detectable products, decrease of substrate <20% (“+”), moderate conversion—decrease of substrate <50% (“++”), good conversion—decrease of substrate >50% (“+++”);

* introduced (green) and released functional group (red);

** *not in the EPA priority pollutants list; “nt” not tested”; P450, cytochrome; P-450 monooxygenase; DIOx, Rieske-type dioxygenase; LAC, laccase; TYR, tyrosinase; LIP, lignin peroxidase; MNP, manganese peroxidase; VP, versatile peroxidase; HRP, horseradish peroxidase; CPO, chloroperoxidase; DYP, dye-decolorizing peroxidase; LPO, lactoperoxidase; BMM, bacterial multicomponent monooxygenase; FMO, flavin-dependent monooxygenase (flavoprotein monooxygenase)*.

### Chlorobenzene and its derivatives

**Figure 2:**
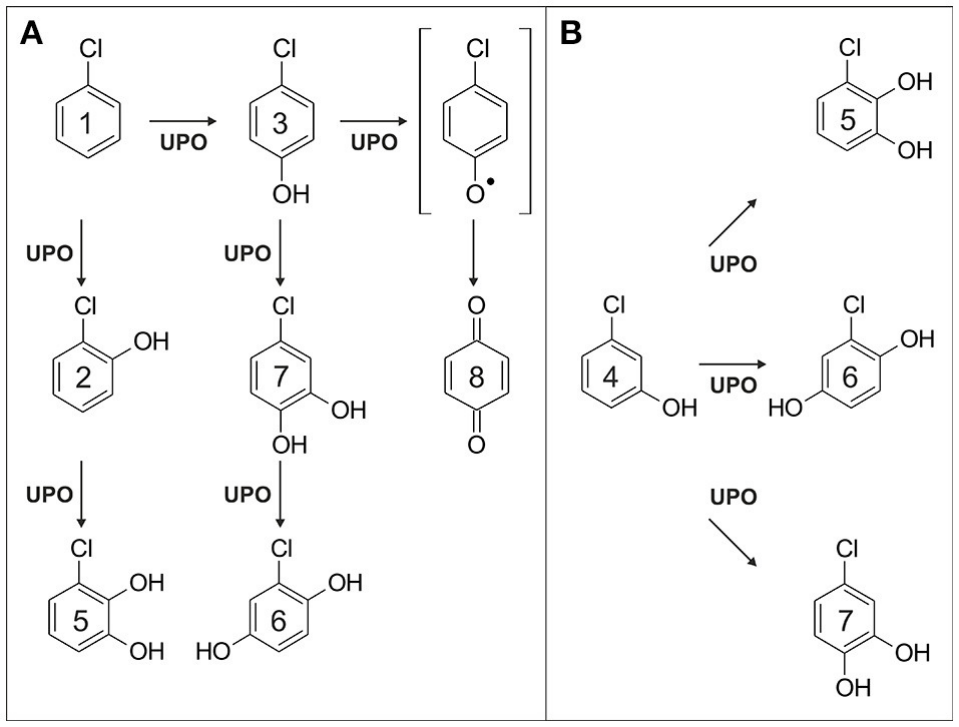
Proposed reaction scheme of chlorobenzene (scheme **A**, 1) and 3-chlorophenol oxidation (scheme **B**; 4) catalyzed by *Aae*UPO; formation of the phenoxy radical is postulated.

Chlorobenzene (**1**) was oxygenated by *Aae*UPO to give 2-chlorophenol (**2**) and 4-chlorophenol (**3**) as major products; oxygenation at the *meta* position of **1** and thus formation of 3-chlorophenol (**4**) was not observed. Further oxidation of **2** led to 3-chlorocatechol (**5**) and chlorohydroquinone (**6**), whereas oxidation of **3** gave 4-chlorocatechol (**7**); **5**, **6**, and **7** are direct oxygenation products of **2**, **3**, or **4**, respectively (Figures 2A,B). *p*-Benzoquinone (**8**) was detected (Figure 2A) when ascorbic acid was omitted from the reaction mixture of **3**; *vice versa*, **8** was not observed in the presence of ascorbic acid. Hence **8** must be a product deriving from two consecutive or parallel enzymatic one-electron oxidations, which represents a type of oxidative dehalogenation known from LAC and POX (Hammel and Tardone, 1988; Osborne et al., 2007; Kordon et al., 2010). Hydrogen abstraction at the phenolic function of **3** would give a phenoxy radical. Two of the latter can disproportionate to **3** and an arene cation (Ullrich and Hofrichter, 2007). A nucleophile, e.g., water, may add to the aromatic cation and subsequent elimination of hydrochloric acid leaves **8** behind, analogously to the Ritter reaction (Krimen and Cota, 2004). The pathway described resembles the enzymatic dehalogenation of **3** described for dehaloperoxidases (Osborne et al., 2009). Masses of triple hydroxylated products arising from **5** to **7** were detected in low amounts; however, their unambiguous identification was not possible, due to the lacking of authentic standards.

Oxygenation of three dichlorobenzenes was indicated by the detection of the corresponding dichlorophenols in the reaction mixture. In the case of 1,3-dichlorophenol, dehalogenation occurred in a second step upon oxygenation giving rise to chlorohydroquinone. The reaction cascade is assumed to proceed analogously to the dehalogenation of **3**. Dehalogenation products were also observed when *p*-chloro-*m*-cresol and 2,4,6-trichlorophenol were applied as UPO substrates.

Interestingly, the conversion of chlorinated benzenes did not follow the expected reaction sequence; thus the introduction of chlorine substituents usually decreases the charge density of the aromatic system and hence inactivates the latter. However, all three dichlorobenzenes and 1,2,3-trichlorobenzene where more effectively oxidized by *Aae*UPO than **1**. A possible explanation for that finding could be the “steric fixation” of the substrate molecule inside the heme pocket, positively affected by two or more chlorine substituents, resulting in a closer distance to the enzyme's reactive compound I and/or less motion within the heme pocket. To our best knowledge, only P450s and DIOX have been reported to oxygenate mono- and dichlorinated benzenes (de Bont et al., 1986; Spiess et al., 1995; Nedelcheva et al., 1998; Jones et al., 2001; Monferran et al., 2007).

All three tested chlorophenols were oxygenated by *Aae*UPO. This was most evident when ascorbic acid was present in the reaction mixtures, which prevented polymerizing side activities. Chlorocatechols (i.e., **5** and **7** and not chlorohydroquinones) were the major products deriving from the oxygenation of **3** and **4**. This is an interesting fact, since chlorocatechols are the substrates of ring-cleaving DIOX within intracellular degradation pathways of chlorinated arenes (Kaschabek et al., 1998; Moiseeva et al., 2002). Thus, we can consider UPOs being involved in fungal catabolic routes of chloroaromatics, with the advantage that toxic chlorophenols will not have to be taken up into the hyphae (Mars et al., 1997). Chlorophenols and chlorocatechols can additionally serve as substrates for one-electron oxidations and thus, besides oxygenases (Beadle and Smith, 1982; Xu and Bhandari, 2003), several POX and phenol oxidases (LAC, TYR) were found to oxidize chlorinated phenols and their derivatives to reactive phenoxy radicals (Xu and Bhandari, 2003; Zhang et al., 2008; Hibi et al., 2012).

Neither oxygenation nor one-electron oxidation was observed when hexachlorobenzene (HCB) and pentachlorophenol (PCP) were applied as substrates for *Aae*UPO and *Mro*UPO. They are the only halogenated compounds tested here that were not converted. Chlorine substituents in higher number may protect the arene C-atoms from attack by UPO's compound I via steric hindrance and/or the impossibility to find a suitable point of attack. On the other hand, some P450s were found to be able to oxygenate both HCB and PCP (Jones et al., 2001; Crawford et al., 2007), and the phenolic functionality of PCP makes it susceptible to one-electron oxidation catalyzed by phenol oxidases and POX (Reddy and Gold, 2000; Montiel et al., 2004; Jeon et al., 2008; Fodil et al., 2011). All other tested halogenated compounds (compare Table 1) served also as substrates for UPOs, but are not explicitly discussed here; more pieces of information are given in the Supplementary section.

Benzoquinones and polymerization products emerged in all reaction setups where ascorbic acid was omitted. The latter acted as radical scavenger that reduced chlorinated phenoxy radicals formed via one-electron oxidation (peroxidative activity of UPO) and prevented that way radical coupling (Niki, 1991).

### Nitroarenes

**Figure 3:**
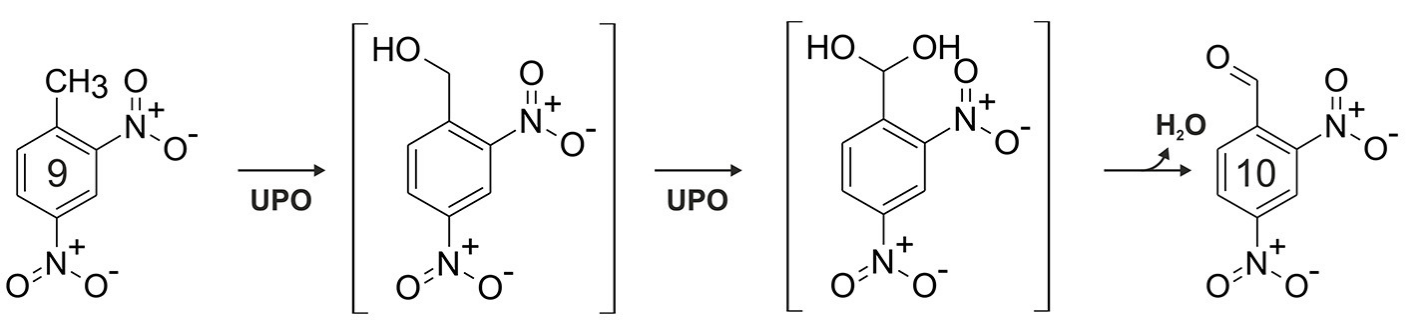
Proposed oxidation sequence for 2,4-dinitrotoluene (**9**) leading to 2,4-dinitrobenzaldehyde (**10**).

The charge density at the aromatic ring is reduced by nitro substituents; thus, with regard to electrophilic attack, nitroarenes are strongly deactivated compounds (McDaniel and Brown, 1958; Spain, 1995). This property is reflected by the low reactivity of UPOs toward nitroaromatic compounds (compare Table 1) and consequently, oxygenation of nitrobenzene was not observed. On the other hand, 2-nitrophenol and 4-nitrophenol served as substrates and were oxidized into the corresponding dihydroxylated nitrobenzenes, which in turn underwent one-electron oxidation resulting in the formation of coupling products. A second nitro group (e.g., 2,4-dinitrophenol), however, made an enzymatic attack by UPOs impossible. Trace amounts of oxidation products were found when 2,4-dinitrotoluene (**9**) and 4,6-dinitro-*o*-cresol were applied as substrates. Since the electron density at the aromatic ring of **9** is lower than in 2,4-dinitrophenol, it is assumed that hydroxylation took place at toluene's methyl group, which in case of 2,6-dinitrotoluene is shielded by two flanking nitro groups preventing attack by UPO compound I. A second indication for the oxidation at the benzylic carbon of **9** is the mass shift of “+14” for one of the products detected, which cannot be explained by aromatic ring hydroxylation. In consequence, we conclude that **9** was attacked by two consecutive two-electron oxidations (compare Figure 3) resulting in the formation of 2,4-dinitrobenzaldehyde via the corresponding benzyl alcohol and *gem*-diol (aldehyde hydrate) intermediates. This finding confirms similar observations previously made for 4-nitrotoluene oxidation by *Aae*UPO (Kinne et al., 2010).

Overall, these results are not surprising when considering literature data of other enzymes. Only a few oxidoreductases are able to oxidize nitrobenzene, e.g., a few DIOXs and BMMs (Spain, 1995; Fishman et al., 2004). The latter was also found to oxidize nitrophenols (Fishman et al., 2004). Furthermore, oxygenation of nitrophenols was reported for some P450s and FMOs (Cassidy et al., 1999; Ye et al., 2004), whereas one-electron oxidation of nitrophenols can be realized by high-redox potential POX, e.g., DYP (Büttner et al., 2015). In contrast, reductive pathways for nitroaromatics are widely distributed in nature and have been well summarized in previous reviews (Spain, 1995; Ye et al., 2004).

### Phthalate esters

In the course of a screening, six phthalate esters (PEs) were tested for oxygenation by *Aae*UPO and *Mro*UPO and three of them were converted: butyl benzyl phthalate, di-*n*-butyl and di-*n*-octyl phthalate. In case of the latter, only trace amounts of products could be detected. Analogously to 2,4-dinitrotoluoene, the “+14” mass shift of products rules out that the oxygen insertion occurred at the aromatic ring and thus oxygenation at the β-position of the alkyl moieties (i.e., butyl or octyl) is most plausible (Peter et al., 2011). No conversion was observed for *bis*(2-ethyl-hexyl) phthalate and the short chain PEs, such as dimethyl phthalate and diethyl phthalate.

Most studies dealing with the degradation of PE have used whole cells (bacterial or fungal pure or mixed cultures). Hydrolysis of the ester bond by esterases was the typical reaction observed (Wang et al., 1995, 2003; Staples et al., 1997). The ring of phthalic acid can be oxidized by a specific bacterial DIOX resulting in the formation the corresponding catechol (Eaton and Ribbons, 1982); side chain oxidation of PEs was also reported for human PE metabolism probably realized by liver P450s (Wittassek and Angerer, 2008). Wittassek and Angerer (2008) reported the oxidation of long chain PE, e.g., di(2-ethyl-hexyl) phthalate, by a P450 and emphasized that short chain PEs, (e.g., dimethyl and diethyl) phthalate, were not oxidized by this enzyme; an observation that corresponds with our findings here.

### Polycyclic aromatic hydrocarbons

Eleven PAHs were tested for the conversion by *Aae*UPO and *Mro*UPO. The majority of them was in fact oxygenated and oxidized by both UPOs with the exception of bulky dibenz[a,h]anthracene and benzo[g,h,i]perylene; benzo[b]fluoranthene was a substrate for *Mro*UPO only. In dependence on the particular PAH, the extent of product formation reached from trace amounts (e.g., benzo[k]fluoranthene) to substantial amounts (e.g., acenaphthylene).

**Figure 4:**
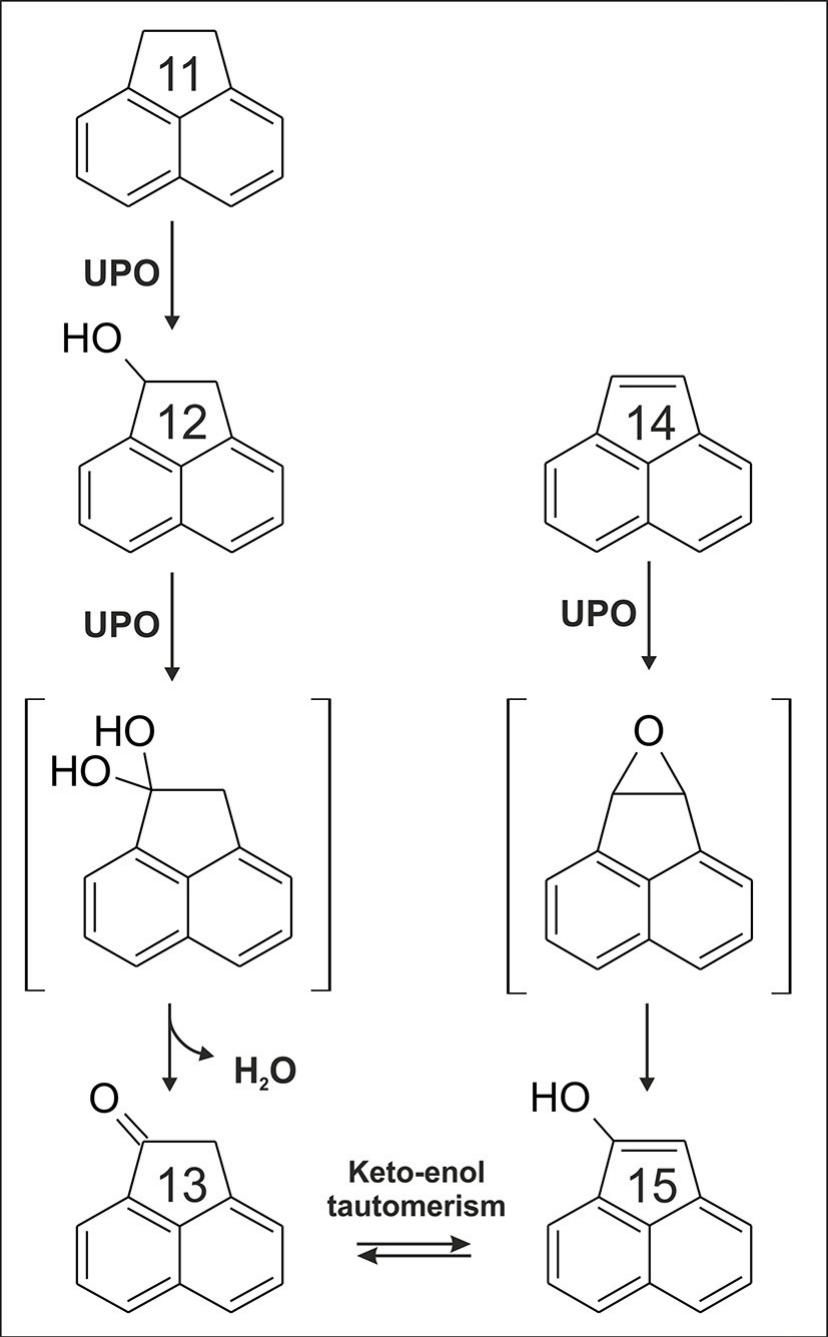
Proposed reaction sequence for the formation of acenaphthenone (**13**) deriving from acenaphthene (**11**) or acenaphthylene (**14**).

From all PAHs tested, acenapthylene (**14**) was oxidized to the highest extent by both UPOs. The major product detected was a monohydroxylated metabolite (m/z +16) with a UV-Vis spectrum resembling that of 1-naphthol (Kluge et al., 2009) (data in suplement). Hence, we assume that oxygenation of **14** proceeded via a 4,5-epoxy acenaphthylene intermediate to give 5-hydroxy acenaphthylene, analogously to naphthalene oxygenation catalyzed by UPO (Kluge et al., 2009). Another interesting finding was the detection of acenaphthenone (**13**) in the reaction setup of **14**. Oxygenation of **14** led to 1-hydroxy-acenaphthylene (**15**) that is the enolic form of **13**. Again, an epoxide intermediate can be postulated, i.e., 1,2-epoxy acenapthene (compare Figure 4).

Thirteen **(13)** was also a product deriving from acenaphthene (**11**) that was next to **14** the best PAH substrate. Oxygenation of a sp^3^-carbon (aliphatic carbon) may give 1-acenaphthol (**12**); via a second attack at the same carbon (i.e., at C1 position), a geminal diol intermediate (*gem*-diol, carbonyl hydrate) may be formed that is in equilibrium via spontaneous dehydration with the corresponding ketone (**13**, Figure 4). Similar to **14**, oxygenation at the aromatic system of **11** was observed as well. However, in contrast to the reaction setup of **14**, two oxygenation products were detected with UV-Vis spectra resembling those of 1-naphthol and 2-naphthol (Kluge et al., 2009). The reported oxidation pathway of P450s for **11** and **14** is rather similar to the reaction sequences proposed herein, including the proof of 1,2-epoxy-acenapthene formation (only the formation of **13** was not ascertained for P450s) (Shimada et al., 2015). Conversion of all other (positively) tested PAHs was evident by detection of products with mass shifts of +16 or +32, representing mono- and dihydroxylated products, respectively.

Water solubility and thus bioavailability decreases with increasing size of PAHs and this fact was reflected by their decreasing relative conversion by UPOs the bigger they were (compare Table 2). It has to be noted that the two UPOs tested accomplished the formation of different PAH products and patterns; thus, *Mro*UPO was capable of hydroxylating more bulky PAHs than *Aae*UPO. This can be explained by the wider heme channel of *Mro*UPOs (11 Å) compared to the relatively narrow channel of *Aae*UPO (7 Å) (Poraj-Kobielska, 2013; Piontek et al., 2017), which limits *AaeU*PO to oxidize PAHs that are larger than 6 Å in diameter (e.g., dibenz[a,h]anthracene or benzo[g,h,i]perylene). The results of the UPO-catalyzed conversion of selected PAHs in relation to some physicochemical properties are summarized in Table 2. Aranda and coworkers had already reported about the successful conversion of several PAHs and related compounds by *Aae*UPO in 2010 (Aranda et al., 2010); four of these PAHs are also listed in Table 2, namely anthracene, fluorene, phenanthrene, and pyrene.

**Table 2:** Conversion of selected PAHs by *Aae*UPO and *Mro*UPO with reference to their water solubility and molecular size.

**Substrate**	**Water solubility [mg L^−1^] (ref.)**	**Carbon atoms [C_n_]**	Minimum and maximum width [Å]^*^	**Relative conversion by *Aae*UPO^#^ (Ref.)**	**Relative conversion by *Mro*UPO**	**Ionization potential [eV] (Ref.)**
Acenaphthylene	16.1 (Tegge, 1983)	12	4.0/4.6	+++	+++	8.22 (Majcherczyk et al., 1998)
Acenaphthene	3.93 (Mackay and Shiu, 1977)	12	4.0/4.6	++	++	7.86 (Majcherczyk et al., 1998)
Fluorene	1.98 (Mackay and Shiu, 1977)	13	3.0/7.4	+++ (Aranda et al., 2010)	n.t	7.89 (Majcherczyk et al., 1998)
Phenanthrene	1.29 (Mackay and Shiu, 1977)	14	4.0/7.6	++ (Aranda et al., 2010)	n.t	7.91 (Majcherczyk et al., 1998)
Anthracene	0.073 (Mackay and Shiu, 1977)	14	3.0/7.9	++ (Aranda et al., 2010)	n.t	7.43 (Majcherczyk et al., 1998)
Pyrene	0.135 (Mackay and Shiu, 1977)	16	5.2/7.6	++ (Aranda et al., 2010)	n.t	7.43 (Majcherczyk et al., 1998)
Benzo[a]anthracene	0.014 (Mackay and Shiu, 1977)	18	4.8/8.1	++	+	7.44 (Majcherczyk et al., 1998)
Benzo[a]pyren	0.0038 (Tegge, 1983)	20	4.8/8.1	+	++	7.12 (Majcherczyk et al., 1998)
Perylene	0.0004 (Mackay and Shiu, 1977)	20	4.6/6.6	t	+	6.97 (Majcherczyk et al., 1998)
Indeno[1,2,3-cd]pyrene	0.062 (Tegge, 1983)	22	5.8/8.4	t	t	–
Benzo[b]fluoranthene	0.0012 (Tegge, 1983)	20	4.8/9.6	0	t	7.70 (Majcherczyk et al., 1998)
Benzo[k]fluoranthene	0.00055 (Tegge, 1983)	20	4.6/10.1	t	t	7.48 (Majcherczyk et al., 1998)
Dibenz[a,h]anthracene	0.0005 (Tegge, 1983)	22	6.9/10.6	0	0	7.38 (Dabestani and Ivanov, 1999)
Benzo[g,h,i]perylene	0.00026 (Mackay and Shiu, 1977)	22	6.7/6.9	0	0	7.16 (Simonsick and Hites, 1984)

# *0, no conversion; t, trace amounts of products detected; +, little conversion; ++, moderate conversion; + + +, good conversion (for more explanations, compare legend of Table 1)*.

* *Calculated with PyMOL 1.3 (https://www.pymol.org)*.

Typical enzymes capable of catalyzing the oxygenation of PAHs are P450s and DIOX (Shimada et al., 1989; Pinyakong et al., 2004). Among fungi, especially P450s were shown to directly oxygenate PAHs (van Gorcom et al., 1998). However, PAHs can be also attacked by enzymes catalyzing one-electron oxidations, such as different POX and LAC, whereat their oxidizability will depend on the ionization potential (IP) and the presence of suitable redox mediators (Sack et al., 1997; Majcherczyk et al., 1998; Johannes and Majcherczyk, 2000; Haritash and Kaushik, 2009). The reaction of these enzymes leads, via instable aryl cations and water addition, to the formation of PAH quinones, in particular in the case of 4-ring and 5-ring PAHs with IP <7.7 eV (Hammel, 1995; Steffen et al., 2003). Quinoid products (whose formation would have been indicated by a mass shift of m/z +28), however, were not detected in our study and the main reaction products of 4- and 5-ring PAHs oxidized by UPOs were mono-hydroxylated products. Thus, substantial one-electron oxidation can be ruled out and hence, the oxyfunctionalizations observed had to be the result of true oxygen transfers (and not of water addition) (Hammel et al., 1986). This finding is largely consistent with the data presented by Aranda et al. (2010) for smaller PAHs, although quinones (e.g., anthraquinone) were observed as minor products. When the data presented herein are being compared with literature data, it becomes evident that quinones are detectable in decreasing order beginning with benzene>naphthalene>anthracene>4- and 5-ring PAHs (e.g., benz[a]anthracene and perylene, respectively); hence the formation of quinoid products from arenes, catalyzed by UPOs, is inversely proportional to the size of the aromatic system (Kluge et al., 2007; Aranda et al., 2010; Karich et al., 2013).

Oxidation of PAHs by ligninolytic peroxidases (e.g., MNP, LIP) and LAC is strictly dependent on the substrates' IP and high-molecular mass PAHs, e.g., benzo[g,h,i]perylene or benzo[a]pyrene, were found to be faster oxidized than smaller PAHs, some of which cannot be oxidized at all (e.g., phenanthrene and fluoranthene) (Steffen et al., 2003). In contrast, UPOs favor low-molecular mass PAHs over high-molecular ones as oxygen acceptor and the substrate IP seems to be of minor relevance for the extent of conversion (Table 2).

### Other substrates

Benzidine (**18**), 1,2-diphenylhydrazine (**16**) and 2,4-dimethylphenol do not really fit into the above classification of potential UPO substrates; therefore, we deal with them herein separately. Treatment of **18** samples with *Aae*UPO and *Mro*UPO mainly resulted in the formation of coupling products. However, in the case of *Mro*UPO, an oxygenation product of **18** was detectable as well. Again, the larger heme channel of *Mro*UPO (compared to *Aae*UPO) may explain that fact. We assume that 3-hydroxybenzidine (**19**) was the product formed, which is most plausible to proceed via a lateral oxidative attack on **18**. Azo derivatives based on **18** have been widely used as textile dyes and some of them are known to be carcinogenic (Lowry et al., 1980). Thus, **18** has been subject of various (eco)toxicological studies with focus on its carcinogenicity. In this context, oxygenation/hydroxylation of **18** by P450s as well as its POX-catalyzed oxidation to benzidine diimine ensuing binding to DNA were described (Yamazoe et al., 1988; Lakshmi et al., 1996). To supplement this, we have shown here that UPOs are capable of catalyzing these reactions as well.

**Figure 5:**
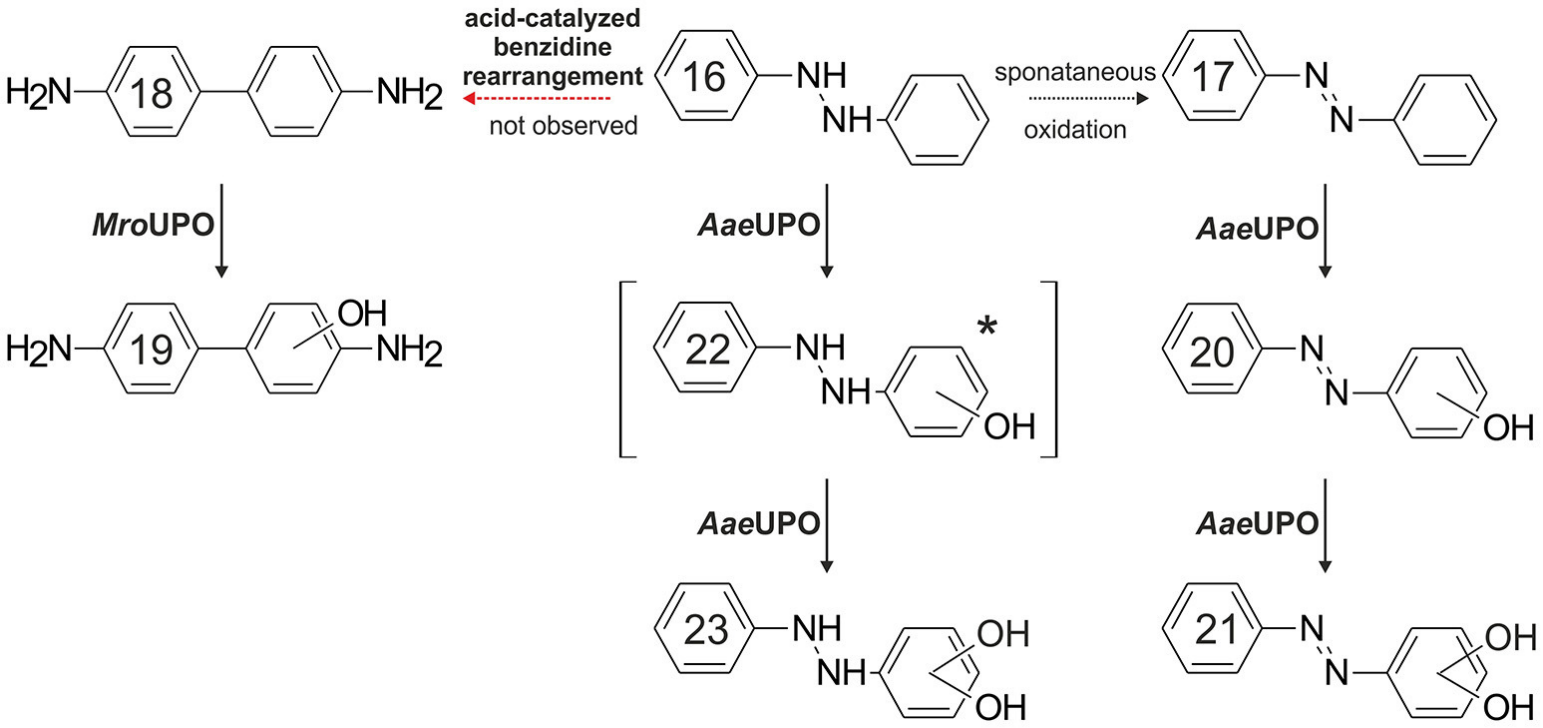
Proposed reaction sequence for the UPO-catalyzed hydroxylation of 1,2-diphenylhydrazine (**16**) and azobenzene (**17**). Detection of a double-hydroxylated 1,2-diphenylhydrazine would require a preceding hydroxylation of **16**; however, a mono-hydroxylated 1,2-diphenylhydrazine (**22**) was not detected [^*^]. (**16**) spontaneously oxidized to *trans*- and *cis*-azobenzene (**17**); subsequent oxygenation catalyzed by *Aae*UPO resulted in single and double hydroxylated azobenzenes (**20** and **21**, respectively). An acid-catalyzed benzidine (**18**) rearrangement of **16** was not observed. Oxygenation of **18** was catalyzed by *Mro*UPO only.

Studying **16-**conversion turned out to be rather difficult for several reasons. The compound instantaneously autoxidizes in aqueous solution to *cis*- and *trans*-azobenzene (**17**) (Riggin and Howard, 1979) or it rearranges to **18** and diphenyline (Hammond and Shine, 1950; Ghigo et al., 2011); the latter reaction, however, is acid-dependent and was therefore not observed under the conditions applied here. Furthermore, authentic standards of potential **16** products are commercially not available and it was impossible to ionize **16** in a way to get a quality mass spectrum. Nevertheless, one product with a mass spectrum shift of “+32” in relation to **16** was detected and might be the result of two hydroxylations at the benzene rings of **16**. Two products most probably deriving from the oxygenation of spontaneously formed **17** were additionally detected. The first product's mass spectrum shifted by m/z+16 in relation to **17** (and by m/z+14 compared to **16**). The latter mass (+14) could hypothetically stand for a single keto function, which is, however, impossible to emerge at the aromatic rings of **16**. Therefore, it may rather represent an oxygenation product of **17** (a hydroxy-azobenzene, **20**). Logically, the second product (mass shift m/z+32 compared to **17**) presumably resulted from a second hydroxylation of **17** (or it may represent a quinone of a double hydroxylated **16**). However, whether *cis*- or *trans*-**17** served as initial substrate, could not be exactly found out but the decrease of *trans*-**17** in samples containing *Aae*UPO (compare Supplementary Material) implies the latter. A proposed pathway for spontaneous and UPO-catalyzed hydroxylation of **16** is given in Figure 5. Mammalian metabolism of **17** and derivatives usually proceeds via reductive pathways and **16** typically rearranges to **18** (Walker, 1970; Levine, 1991). Hydroxylation of **17** or **16** by mammalian P450 was not observed (Bray et al., 1951).

In the reaction setup of 2,4-dimethylphenol with *Aae*UPO, polymerization products, oxygenation products and combinations of both were found. This finding is not surprising, since methylphenols (cresols) are well-known substrates both for one-electron oxidations by POX (or LAC) (Klibanov et al., 1980; Ghosh et al., 2008) and for oxyfunctionalizations by P450s (Yan et al., 2005) and other monooxygenases (Arenghi et al., 2001). Thus, our results fit well to these reports and supplement an own previous study dealing with benzene oxidation by *Aae*UPO, in the course of which phenol emerged as an intermediate that was rapidly further converted (Karich et al., 2013).

### Concluding remarks

The majority of organopollutants tested here (i.e., 35 out of 44 substances, compare Table 1)—including xenobiotics, such as chlorinated benzenes and their derivatives, halogenated biphenyl ethers, nitroaromatic compounds, polycyclic aromatics and phthalates—were oxidatively converted by two fungal model UPOs. UPO-catalyzed oxidations were limited for three main reasons: (i) steric hindrance caused by the number of substituents or general bulkiness of the compound, e.g., hexachlorobenzene or large PAHs, such as benzo[g,h,i]perylene; (ii) strong inactivation of the aromatic ring by electron-withdrawing groups, e.g., nitrobenzene, and (iii) low bioavailability (water solubility) of the potential substrate.

Currently, 41 EPA priority pollutants have been reported to be oxidized by UPOs including the herein tested compounds and several other substances investigated in previous studies. Intensely investigated *Aae*UPO was found to oxygenate and oxidize numerous substance classes (Aranda et al., 2009, 2010; Kluge et al., 2009, 2012; Kinne et al., 2010; Peter et al., 2011; Poraj-Kobielska et al., 2011; Karich et al., 2013; Poraj-Kobielska, 2013) and at present, as much as 300 aromatic, heterocyclic, aliphatic and alicyclic compounds have been identified to serve as substrates for this enzyme (Hofrichter et al., 2015). This fact points out that UPOs are highly versatile oxidoreductases with an exceptional broad substrate spectrum. Besides UPOs, only P450s realize a comparable catalytic promiscuity for oxyfunctionalization reactions (compare Table 1). In fact, UPOs share with P450s the heme-thiolate as prosthetic group as well as highly reactive compound-I and protonated compound-II intermediates in the catalytic cycle (Yosca et al., 2017). On the other hand, UPOs and P450s do not share any sequence homology and act in different micro-environments (extracellularly vs. intracellularly). Maybe the catalytic systems of P450s and UPOs complement each other in a suitable way by eliminating similar, often toxic compounds inside and outside of fungal cells, respectively. Thus, UPOs are secretory enzymes using the rather simple co-substrate (H_2_O_2_) that can be generated outside the fungal hyphae, while P450s—as membrane-bound or cytosolic enzymes—use a complex accessory machinery, which allows their precise action in different hyphal compartments. In other words, UPOs can directly interact with the fungus' micro-environment and do rather “dirty catalytic jobs,” whereas P450s are responsible for the fine-tuning of similar reactions in the cells. UPOs may represent an extracellular equivalent to intracellular P450s, in which they function as a universal fungal detoxification system (“extracellular fungal liver”) that can oxidize plant ingredients, microbial metabolites and xenobiotics. The high number of putative UPO genes distributed among rather different ecological and phylogenetic groups of fungi (compare Figure 1) may strongly support this assumption.

Until now, all substrate conversion studies regarding UPOs have been carried out in cell-free systems with isolated enzyme preparations and it is still unclear, under which circumstances UPOs are induced and expressed in fungi under natural conditions. So it is rather difficult to appraise, which roles (others than detoxification) these enzymes may still play. Whilst the actual physiological functions of UPOs in individual fungi will still have to be elucidated, the wealth of catalyzed reactions is without doubt and in any case, interesting from the environmental and biotechnological points of view. Notably, UPOs do not only complement P450 activities but they may also support the action of extracellular fungal enzyme systems catalyzing one-electron oxidations, as needed for lignin and humus decomposition (i.e., POX and LAC reactions).

Future studies on UPOs will have to focus, amongst others, on the conditions, under which the production and secretion of UPOs are induced and how their activities can be stimulated in different ecological and phylogenetical groups of fungi. Because UPO genes are widely distributed in the whole fungal kingdom and fungi indeed permeate the living scene, a powerful tool may become available to foster bioattenuation processes, such as the self-cleaning function of soils.

## Author contributions

Conceived and designed the experiments: AK, RU, and MH. Performed the experiments: AK. Wrote the manuscript, supervised, and discussed the experiments and data: AK, RU, KS, and MH.

### Conflict of interest statement

The authors declare that the research was conducted in the absence of any commercial or financial relationships that could be construed as a potential conflict of interest.

## Abbreviations

UPO: unspecific peroxygenase
*Aae*: *Agrocybe aegerita*
*Mro*: *Marasmius rotula*
POX: peroxidases
LAC: laccase
TYR: tyrosinase
PAH: polycyclic aromatic hydrocarbons
P450: cytochrome-P450 monooxygenase
BMM: Multicomponent monooxygenase
TMO: toluene 4-monooxygenase
DIOX: dioxygenase
LIP: lignin peroxidase
MNP: manganese peroxidase
VP: versatile peroxidase
HRP: horseradish peroxidase
DYP: dye-decolorizing peroxidase
CPO: chloroperoxidase
HCB: hexachlorobenzene
PCP: pentachlorophenol
PE: phthalate esters
IP: ionization potential.
