# Supplementary material for: Fungal Unspecific Peroxygenases Oxidize the Majority of Organic EPA Priority Pollutants
Source: Front Microbiol. 2017 Aug 9;8:1463. doi: 10.3389/fmicb.2017.01463 (PMC5552789; doi:10.3389/fmicb.2017.01463)
Supplement: Supplementary file 1 [file DataSheet1.PDF]

Supplementary Table 1: Relative conversion of compounds tested as UPO substrates, including type of oxyfunctionalization; * by mass-shift and/or authentic standards; ** not listed under EPA priority pollutants; nt - not tested; compunds that were not attacked by UPOs are marked in red.

#	compound	polymerization	Inserted and deleted functional groups*	Rel. conversion *Aae*UPO	Rel. conversion *Mro*UPO
1	Chlorobenzene	-	-OH =O	+	nt
2	2-Chlorophenol	+	-OH =O	+++	nt
3	1,2-dichlorobenzene	-	-OH	+	nt
4	1,3-Dichlorobenzene	-	-OH -Cl	++	nt
5	1,4-Dichlorobenzene	-	-OH	+	nt
6	2,4-Dichlorophenol	+	-OH	+	+++
7	1,2,4-Trichlorobenzene	-	-OH	++	nt
8	2,4,6-Trichlorophenol	+	-Cl > -OH	+++	nt
9	Pentachlorophenol	-	-	0	0
10	Hexachlorobenzene	-	-	0	0
11	*para*-Chloro-*meta*-cresol	+	-OH -Cl	+++	nt
12	2-Chloronaphthalene	-	-OH	+++	nt
13	3,3-Dichlorobenzidine	+	-OH	++	nt
14	4-Chlorophenyl phenyl ether	-	-OH	++	nt
15	4-Bromophenyl phenyl ether	-	-OH	++	nt
16	3-Chlorophenol**	+	-OH =O	+++	nt
17	4-Chlorophenol**	+	-OH =O -Cl	+++	nt
18	Nitrobenzene	-	-	0	nt
19	2-Nitrophenol	+	-OH	+	nt
20	4-Nitrophenol	+	-OH	++	nt
21	2,4-Dinitrophenol	-	-	0	nt
22	2,4-Dinitrotoluene	-	=O	t	nt
23	2,6-Dinitrotoluene	-	No mass?	t	nt
24	4,6-Dinitro-*o*-cresol	+	-OH	t	nt
25	Benzidine	+	-OH	t	+
26	1,2-Diphenylhydrazine (3 compounds in control!)	-	-OH	++	nt
27	*bis*(2-Ethylhexyl) phthalate	-	-	0	0
28	Butyl benzyl phthalate	-	-OH =O	+	t
29	di-*N*-Butyl Phthalate	-	-OH =O	+	+
30	di-*n*-Octyl phthalate	-	-OH =O	t	t
31	Diethyl Phthalate	-	-	0	0
32	Dimethyl phthalate	-	-	0	0
33	Acenaphthylene	-	-OH =O	++	++
34	Acenaphthene	-	-OH =O	++	++
35	Benzo(a)pyrene	-	-OH	+	++
36	Benzo[a]anthracene	-	-OH	++	+

37	Indeno[1,2,3-cd]pyrene	-	-OH	t	t
38	Benzo[b]fluoranthene	-	-OH	0	t
39	Benzo[k]fluoranthene	-	-OH	t	t
40	Dibenz[a,h]anthracene	-		0	0
41	Benzo[g,h,i]perylene	-		0	0
42	Perylene	-	-OH	t	+
43	9,10-Dihydrophenanthrene	-	-OH	t	+
44	2,4-Dimethylphenol	+	-OH =O	+++	nt

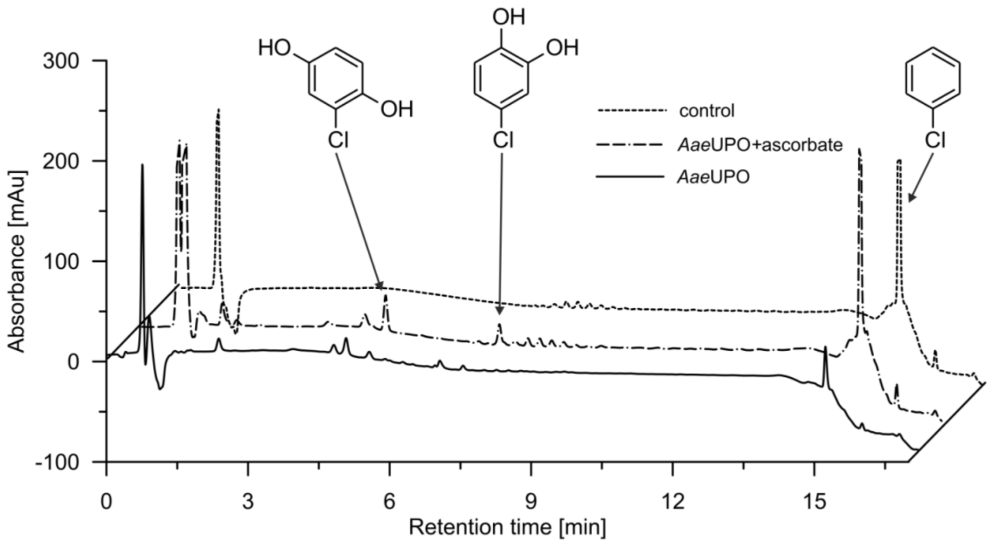

Supplementary figure 1: HPLC-elution profiles (210 nm) of following reaction setup: 0.5 mM Cl-benzene, 1 mM H_2O_2, 0.5 U_{Valk}/mL *Aae*UPO, 20 mM KP_i pH 7, 5% acetonitrile, 4 mM (ascorbate if added). The total reaction volume was 500 µL, addition of H_2O_2 started the reaction. Reaction mixture was analyzed by using **Method 1** after 30 min.

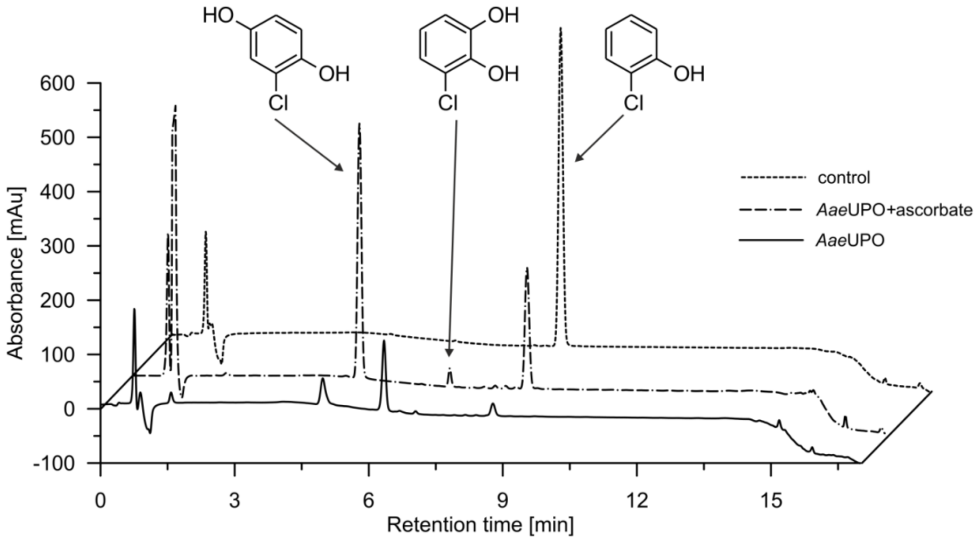

Supplementary figure 2: HPLC-elution profiles (210 nm) of following reaction setup: 0.5 mM 2-Cl-phenol, 1 mM H_2O_2, 0.5 U_{Valk}/mL *Aae*UPO, 20 mM KP_i pH 7, 5% acetonitrile, 4 mM (ascorbate if added). The total reaction volume was 500 µL, addition of H_2O_2 started the reaction. Reaction mixture was analyzed with **Method 1** after 30 min.

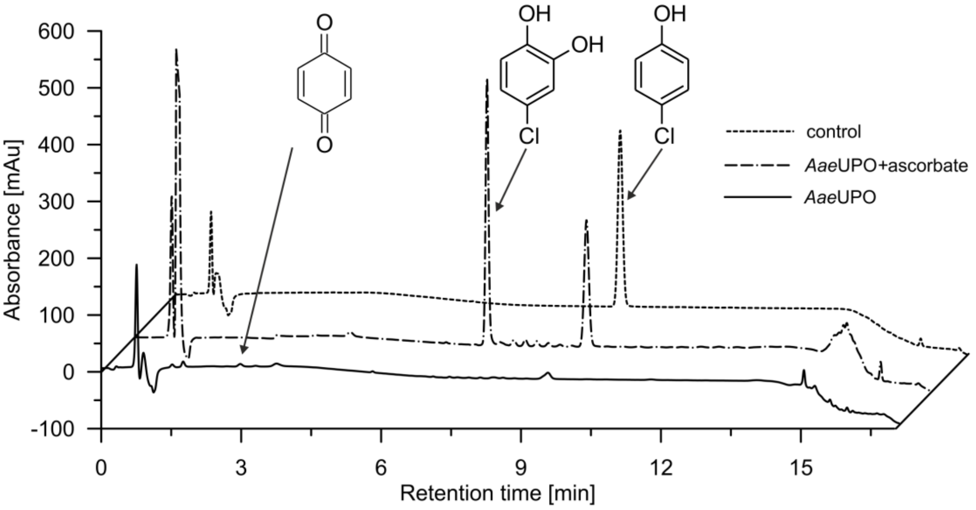

Supplementary figure 3: HPLC-elution profiles (210 nm) of following reaction setup: 0.5 mM 4-Cl-phenol, 1 mM H_2O_2, 0.5 U_{Valk}/mL *Aae*UPO, 20 mM KP_i pH 7, 5% acetonitrile, 4 mM (ascorbate if added). The total reaction volume was 500 µL; addition of H_2O_2 started the reaction. Reaction mixture was analyzed with **Method 1** after 30 min.

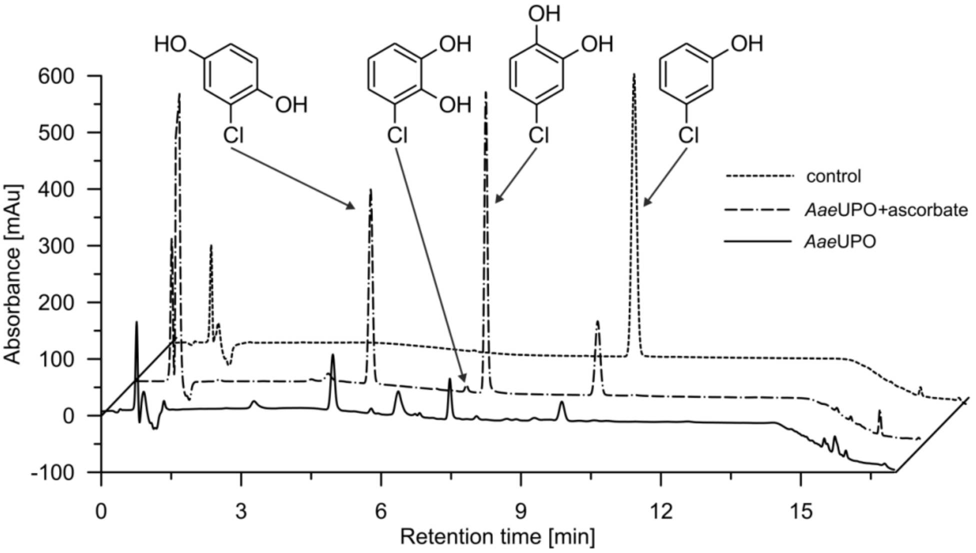

Supplementary figure 4: HPLC-elution profiles (210 nm) of following reaction setup: 0.5 mM 3-Cl-phenol, 1 mM H_2O_2, 0.5 U_{Valk}/mL *Aae*UPO, 20 mM KP_i pH 7, 5% acetonitrile, 4 mM (ascorbate if added). The total reaction volume was 500 µL; addition of H_2O_2 started the reaction. Reaction mixture was analyzed by using **Method 1** after 30 min.

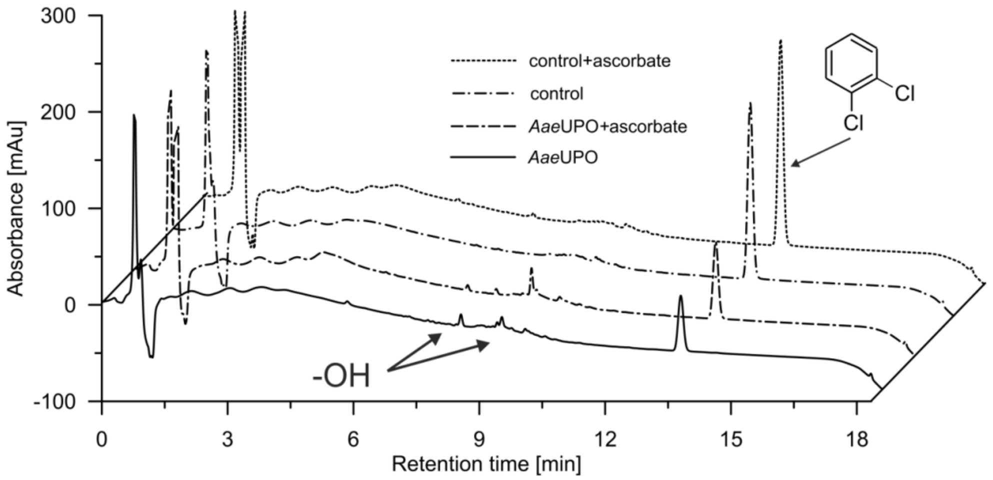

Supplementary figure 5: HPLC-elution profiles (210 nm) of following reaction setup: 0.5 mM 1,2-dichlorobenzene, 1 mM H_2O_2, 0.5 U_{Valk}/mL *Aae*UPO, 20 mM KP_i pH 7, 5% acetonitrile, 4 mM (ascorbate if added. The total volume was 500 µL. H_2O_2 was added via a syringe pump over 30 min. Reaction mixture was analyzed with **Method 2**, 15 min after peroxide addition had been stopped.

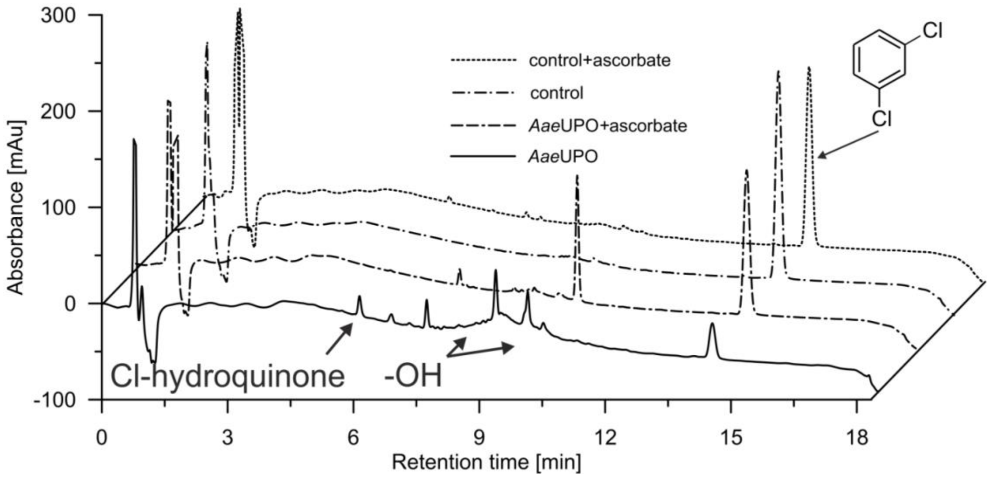

Supplementary figure 6: HPLC-elution profiles (210 nm) of following reaction setup: 0.5 mM 1,3-dichlorobenzene, 1 mM H_2O_2, 0.5 U_{Valk}/mL *Aae*UPO, 20 mM KP_i pH 7, 5% acetonitrile, 4 mM (ascorbate if added). The total volume was 500 µL. H_2O_2 was added via a syringe pump over 30 min. Reaction mixture was analyzed with **Method 2**, 15 min after peroxide addition had been stopped.

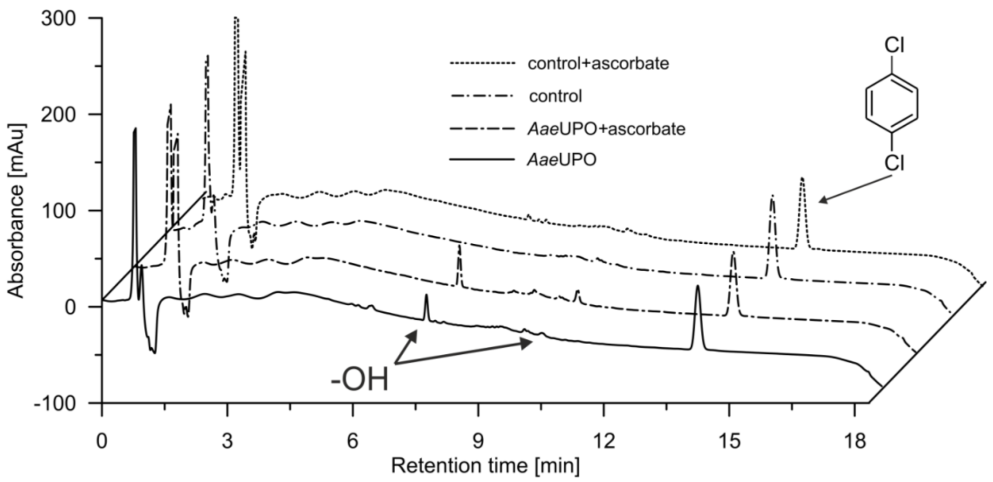

Supplementary figure 7: HPLC-elution profiles (210 nm) of following reaction setup: 0.5 mM 1,4-dichlorobenzene, 1 mM H_2O_2, 0.5 U_{Valk}/mL *Aae*UPO, 20 mM KP_i pH 7, 5% acetonitrile, 4 mM (ascorbate if added). The total volume was 500 µL. H_2O_2 was added via a syringe pump over 30 min. Reaction mixture was analyzed with **Method 2**, 15 min after peroxide additionhad been ceased.

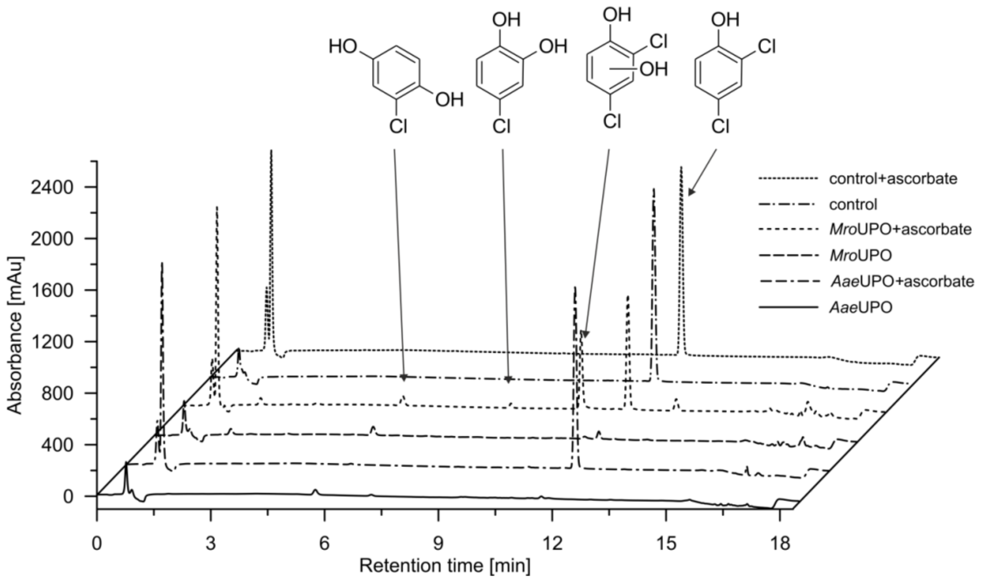

Supplementary figure 8: HPLC-elution profiles (210 nm) of following reaction setup: 0.5 mM 2,4-dichlorophenol, 1 mM H_2O_2, 0.5 U_{Valk}/mL *Aae*UPO/*Mro*Upo, 20 mM KP_i pH 7, 5% acetonitrile, 4 mM (ascorbate if added). The total reaction volume was 500 µL; addition of H_2O_2 started the reaction. Reaction mixture was analyzed with **Method 3** after 30 min.

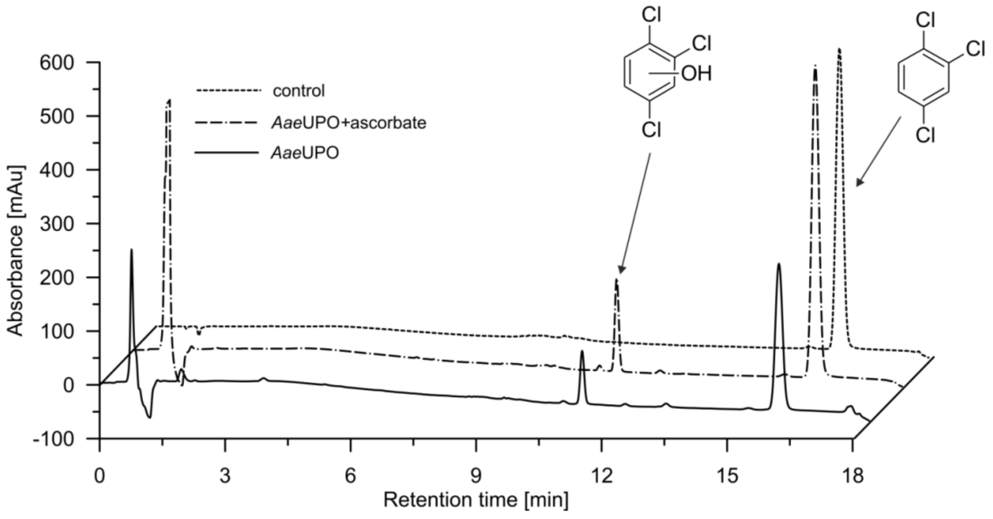

Supplementary figure 9: HPLC-elution profiles (210 nm) of following reaction setup: 1 mM 1,2,4-trichlorobenzene, 1 mM H_2O_2, 1 U_{Valk}/mL *Aae*UPO, 50 mM KP_i pH 7, 10% acetonitrile, 4 mM (ascorbate if added). The total volume was 500 µL. H_2O_2 was added via a syringe pump over 30 min. Reaction mixture was analyzed with **Method 2**,15 min after peroxide addition had been ceased.

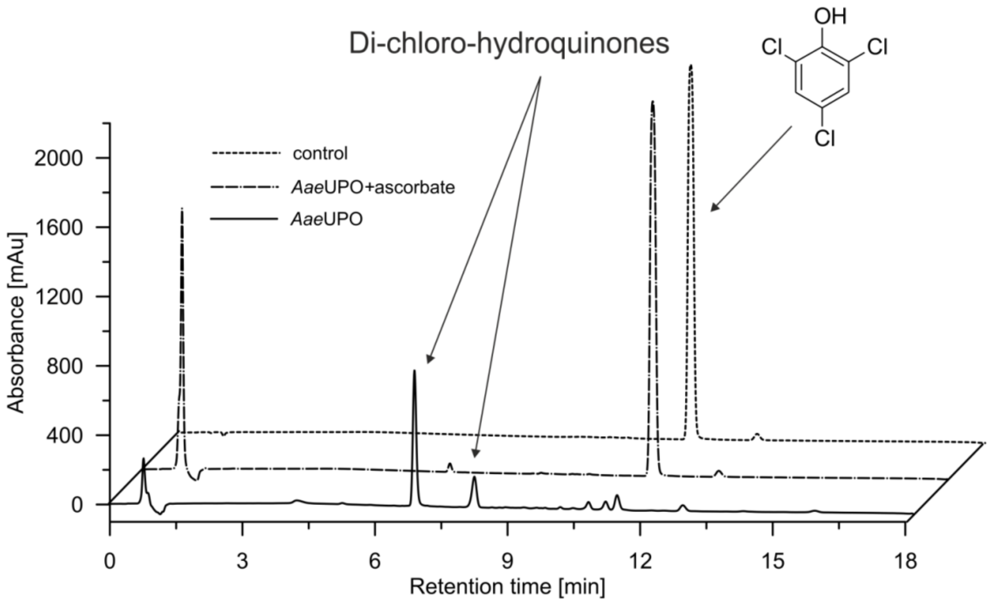

Supplementary figure 10: HPLC-elution profiles (210 nm) of following reaction setup: 1 mM 2,4,6-trichlorophenol, 1 mM H_2O_2, 1 U_{Valk}/mL *Aae*UPO, 50 mM KP_i pH 7, 5% acetonitrile, 4 mM (ascorbate if added). The total reaction volume was 500 µL; addition of H_2O_2 started the reaction. Reaction mixture was analyzed with **Method 2** after 30 min.

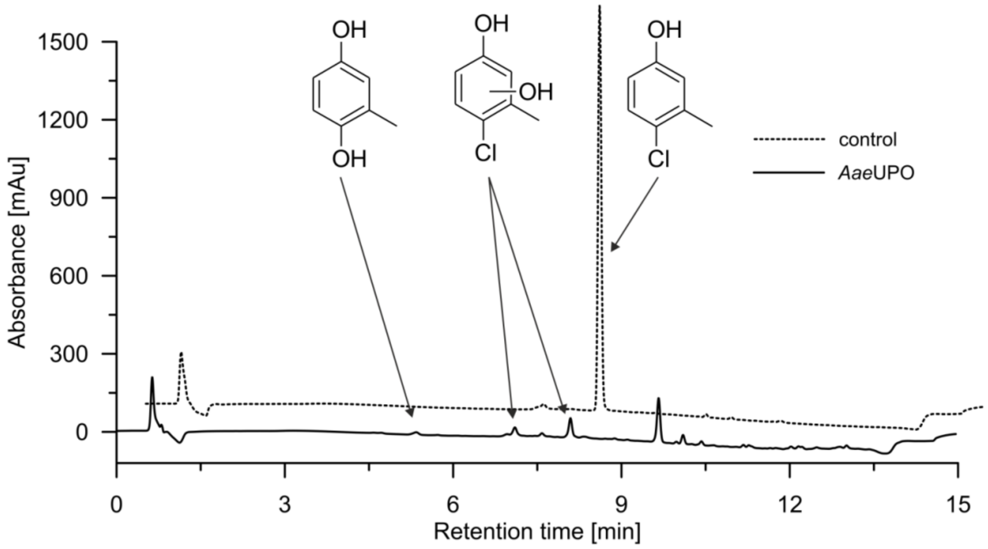

Supplementary figure HPLC-elution profiles (210 nm) of following reaction setup: 0.5 mM *para*-chloro-*meta*-cresol, 1 mM H_2O_2, 0.45 U_{Valk}/mL *Aae*UPO, 50 mM KPi pH 7, 5% acetonitrile, 4 mM (ascorbate if added). Total reaction volume was 500 µL; addition of H_2O_2 started the reaction. Reaction mixture was analyzed with **Method 3** after 30 min.

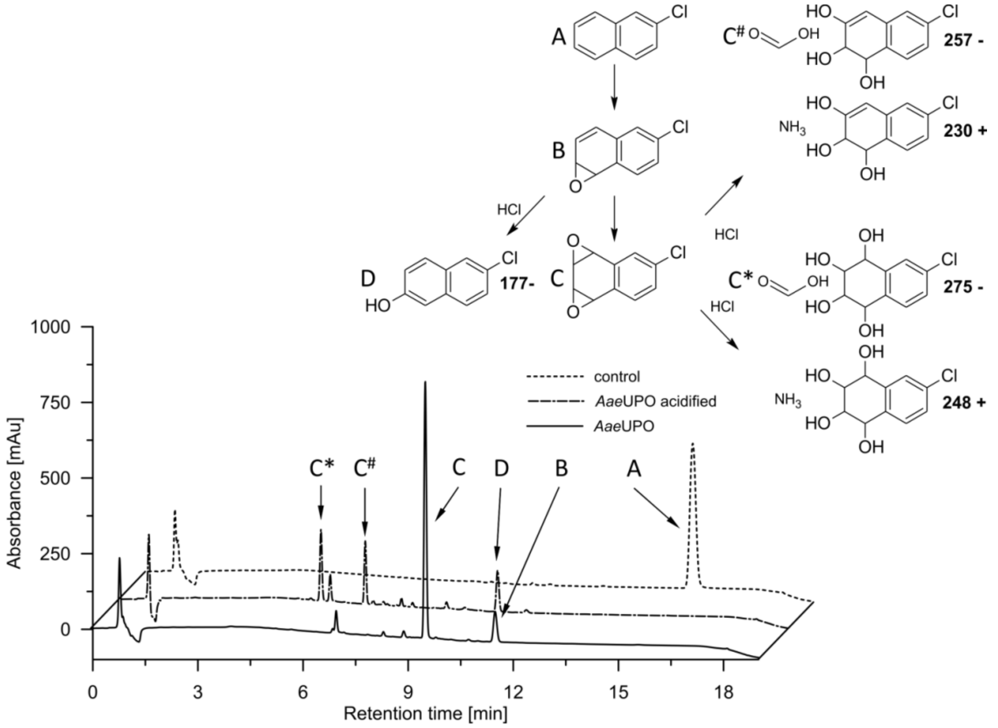

Supplementary figure HPLC-elution profiles (210 nm) of following reaction setup: 0.5 mM 2-chloronaphthalene, 1 mM H_2O_2, 1 U_{Valk}/mL *Aae*UPO, 20 mM KPi pH 7, 5% acetonitrile, 4 mM (ascorbate if added). The total reaction volume was 500 µL; addition of H_2O_2 started the reaction. Reaction mixture was diluted with aecetomnitrile 1:1 (vo/vol) prior to injection into the HPLC system (and in some cases, acidified with 1 µL 1 M HCl). Reaction mixture was analyzed with **Method 2** after 30 min.

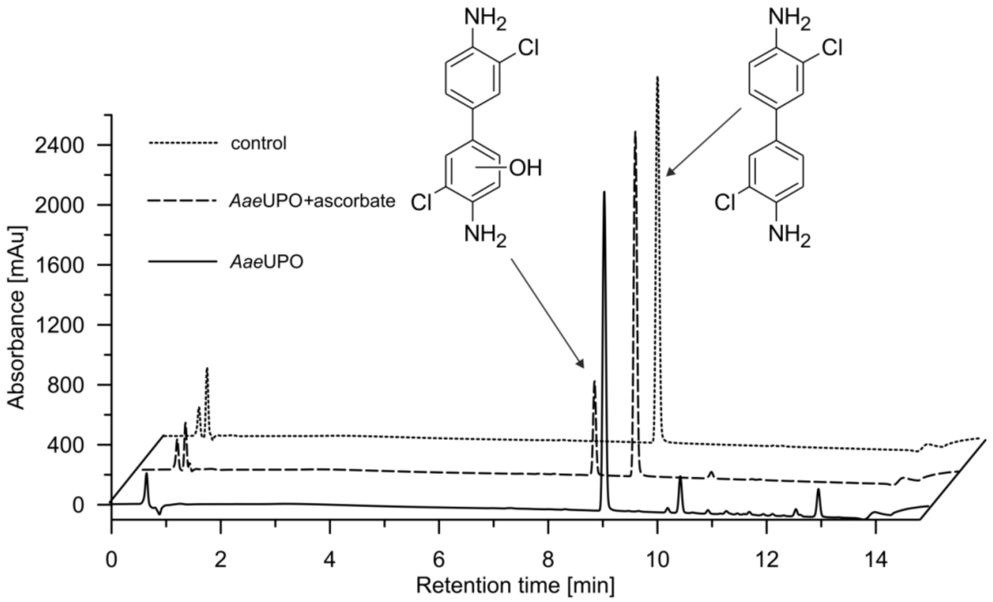

Supplementary figure HPLC-elutionprofiles (210 nm) of following reaction setup: 1 mM 3,3-dichlorobenzidine, 1 mM H_2O_2, 0.45 U_{Valk}/mL *Aae*UPO, 20 mM KPi pH 7, 10% acetonitrile, 4 mM (ascorbate if added). The total reaction volume was 500 µL; addition of H_2O_2 started the reaction. Reaction mixture was analyzed with **Method 4** after 30 min.

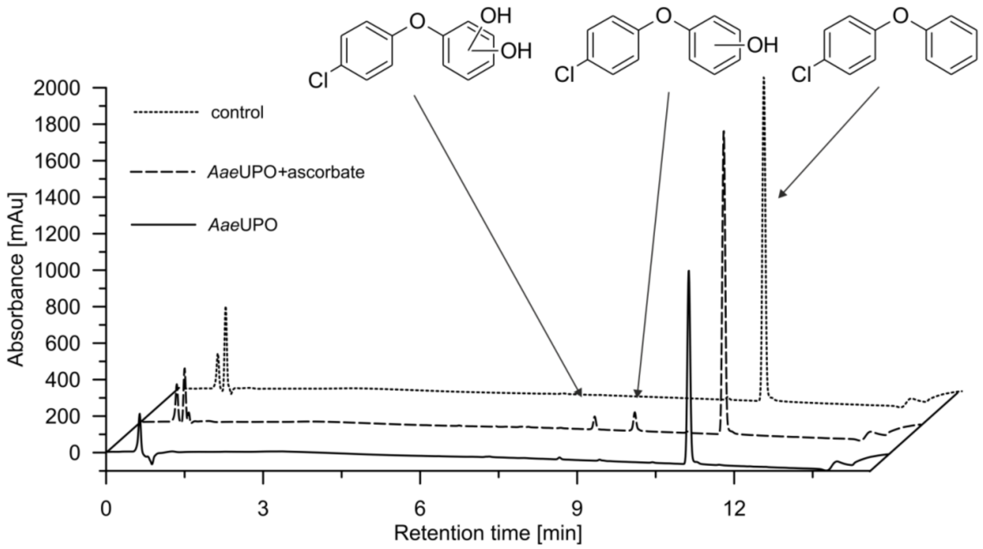

Supplementary figure HPLC-elution profiles (210 nm) of following reaction setup: 1 mM 4-chlorophenyl phenyl ether, 1 mM H_2O_2, 0.45 U_{Valk}/mL *Aae*UPO, 20 mM KPi pH 7, 10% acetonitrile, 4 mM (ascorbate if added). The total reaction volume was 500 µL; addition of H_2O_2 started the reaction. Reaction mixture was diluted with aecetomnitrile 1:1 (vol/vol) prior to injection into the HPLC system. Reaction mixture was analyzed with **Method 4** after 30 min.

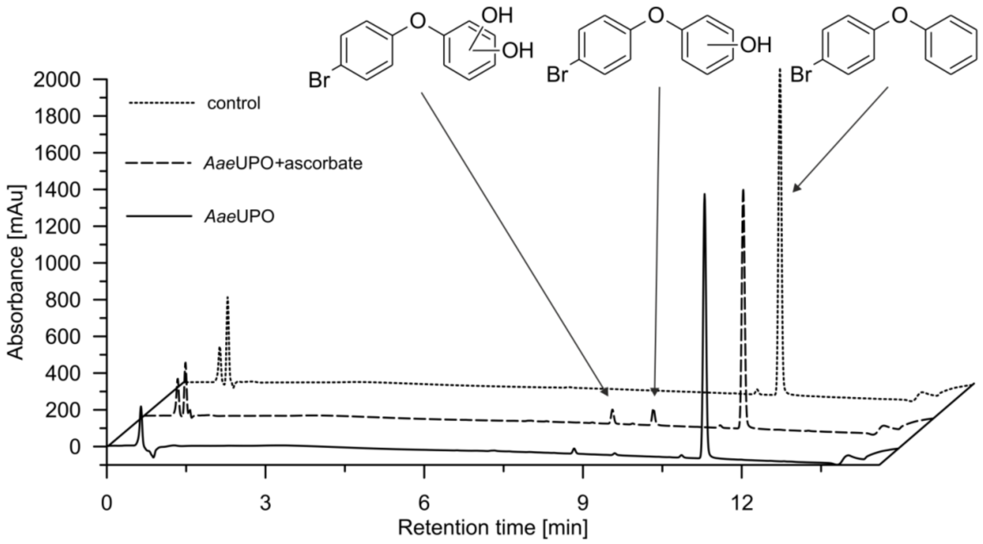

Supplementary figure HPLC-elution profiles (210 nm) of following reaction setup: 1 mM 4-bromophenyl phenyl ether, 1 mM H_2O_2, 0.45 U_{Valk}/mL *Aae*UPO, 20 mM KPi pH 7, 10% acetonitrile, 4 mM (ascorbate if added). The total reaction volume was 500 µL; addition of H_2O_2 started the reaction. Reaction mixture was diluted with aecetonitrile 1:1 (vol/vol) prior to injection. Reaction mixture was analyzed with **Method 4** after 30 min.

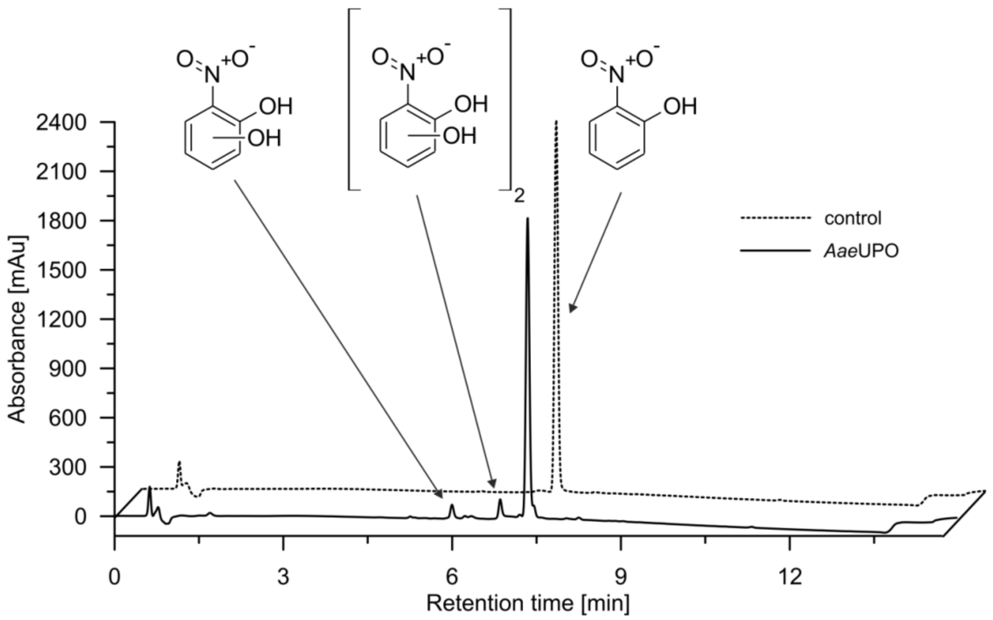

Supplementary figure 16: HPLC-elution profiles (210 nm) of following reaction setup: 1 mM 2-nitrophenol, 1 mM H_2O_2, 0.45 U_{Valk}/mL *Aae*UPO, 50 mM KPi pH 7, 5% acetonitrile. The total reaction volume was 500 µL; addition of H_2O_2 started the reaction. Reaction mixture was analyzed with **Method 3** after 30 min.

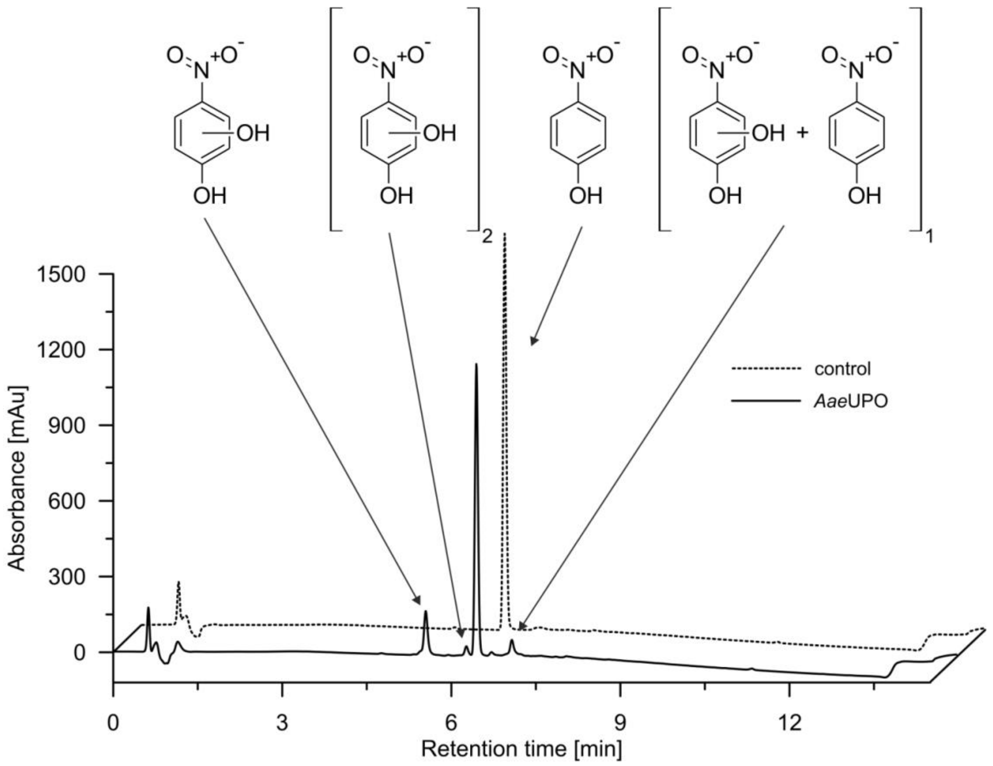

Supplementary figure HPLC-elutionprofiles (210 nm) of following reaction setup: 1 mM 4-nitrophenol, 1 mM H_2O_2, 0.45 U_{Valk}/mL *Aae*UPO, 50 mM KPi pH 7, 5% acetonitrile. The total reaction volume was 500 µL; addition of H_2O_2 started the reaction. Reaction mixture was analyzed with **Method 3** after 30 min.

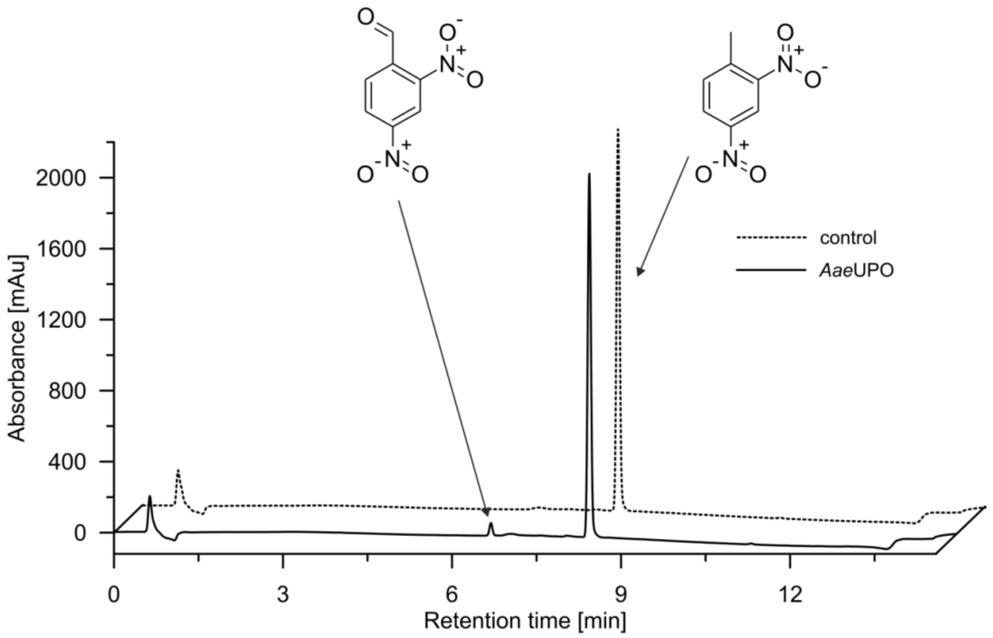

Supplementary figure HPLC-elution profiles (210 nm) of following reaction setup: 1 mM 2,4-dinitrotoluene, 1 mM H_2O_2, 0.45 U_{Valk}/mL *Aae*UPO, 50 mM KPi pH 7, 5% acetonitrile. The total reaction volume was 500 µL; addition of H_2O_2 started the reaction. Reaction mixture was analyzed with **Method 3** after 30 min.

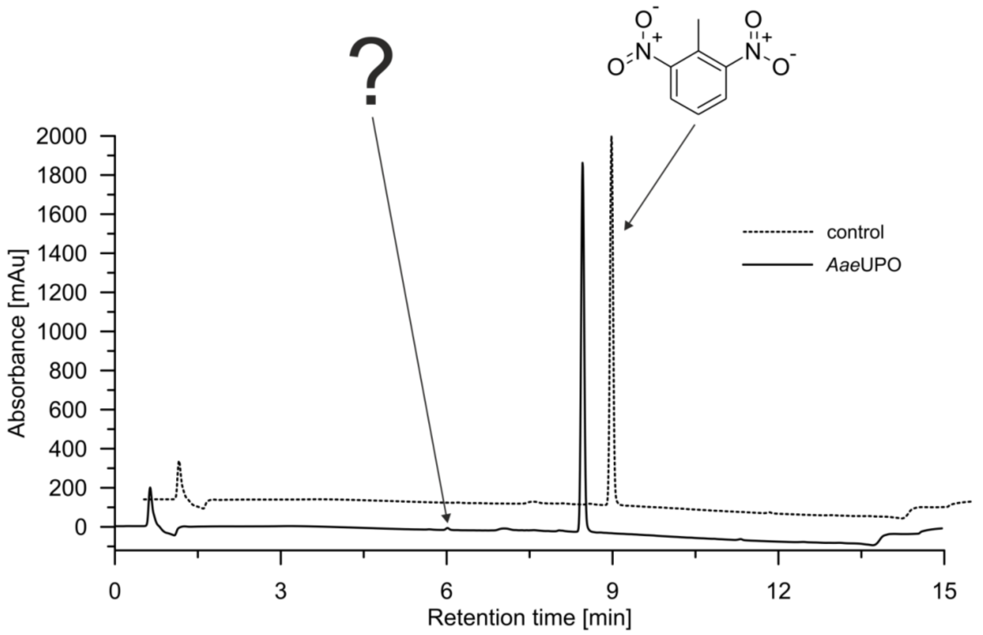

Supplementary figure HPLC-elution profiles (210 nm) of following reaction setup: 2,6-dinitrotoluene, 1 mM H_2O_2, 0.45 U_{Valk}/mL *Aae*UPO, 50 mM KPi pH 7, 5% acetonitrile. The total reaction volume was 500 µL; addition of H_2O_2 started the reaction. Reaction mixture was analyzed with **Method 3** after 30 min.

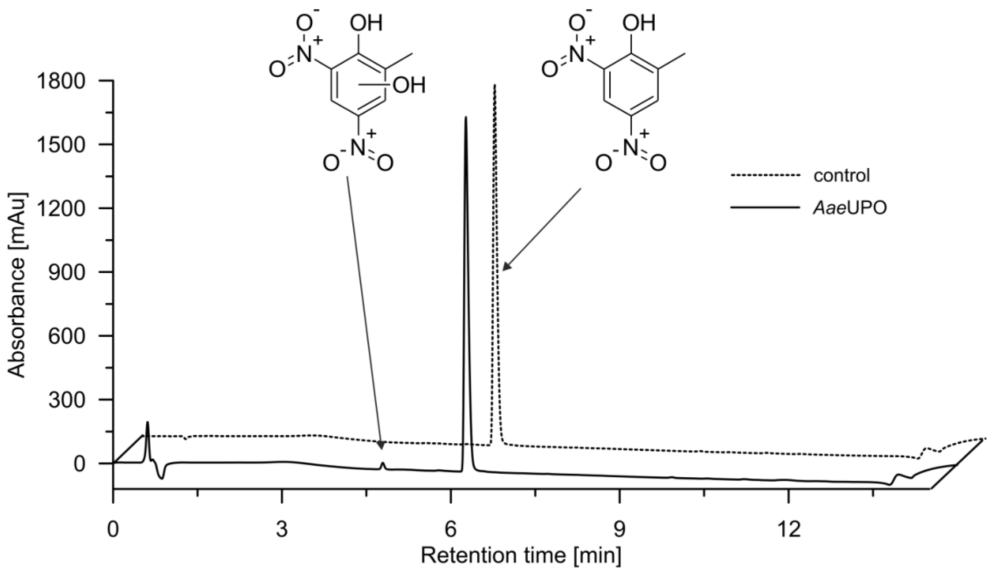

Supplementary figure HPLC-elution profiles (210 nm) of following reaction setup: dinitro-*ortho*-cresol (DNOC), 1 mM H_2O_2, 0.45 U_{Valk}/mL *Aae*UPO, 50 mM KPi pH 7, 5% acetonitrile. The total reaction volume was 500 µL; addition of H_2O_2 started the reaction. Reaction mixture was analyzed with **Method 3** after 30 min.

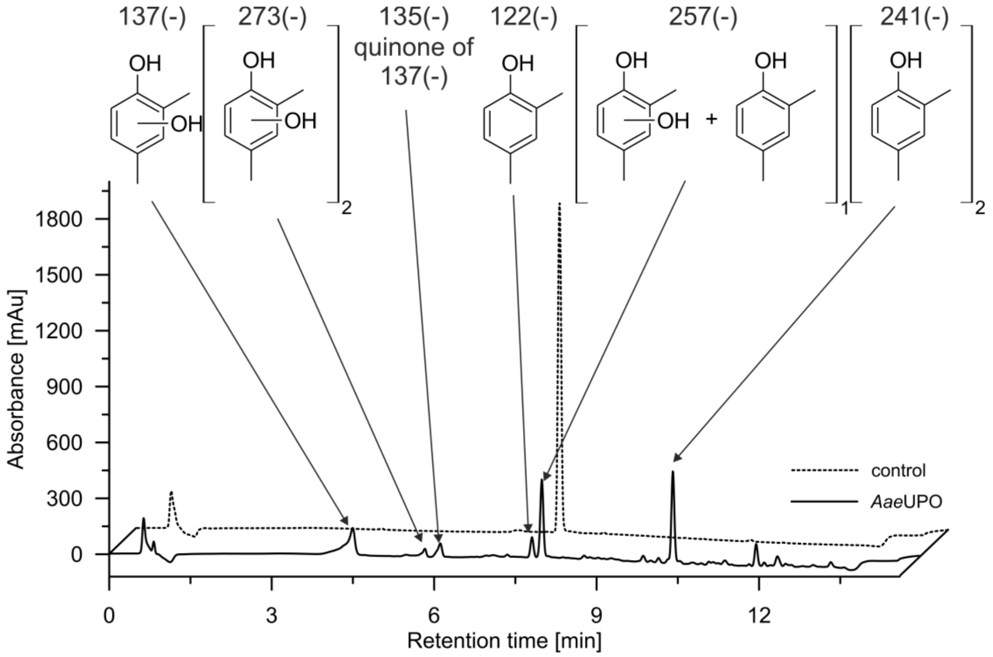

Supplementary figure 21: HPLC-elution profikles (210 nm) of following reaction setup: 1 mM dimethylphenol, 1 mM H_2O_2, 0.45 U_{Valk}/mL *Aae*UPO, 50 mM KPi pH 7, 5% acetonitrile. The total reaction volume was 500 µL; addition of H_2O_2 started the reaction. Reaction mixture was analyzed with **Method 3** after 30 min.

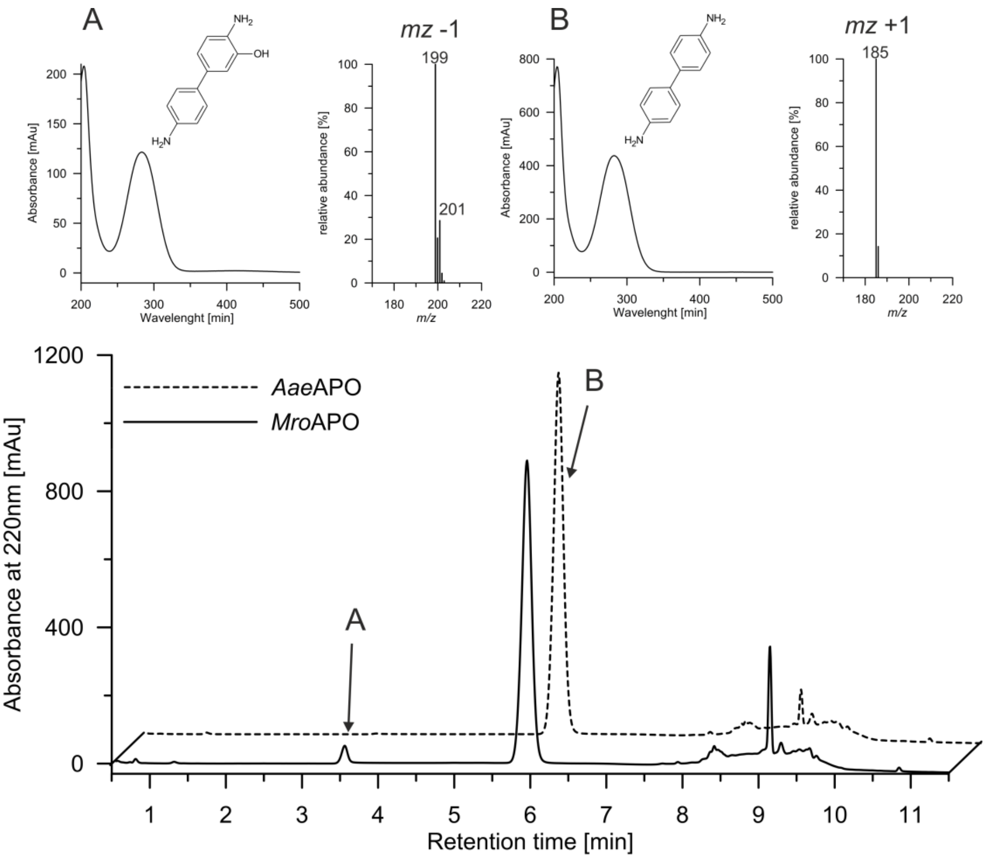

Supplementary figure 22: HPLC-elution profiles (220 nm) of following reaction setup: 1 mM benzidine, 1 mM H_2O_2, 1 U_{Valk}/mL *Aae*UPO/*Mro*UPO, 20 mM KPi pH 7, ascorbic acid 5 mM, 5% acetonitrile. The total reaction volume was 500 µL; addition of H_2O_2 started the reaction. Reaction mixture was analyzed by using **Method 11** after 30 min.

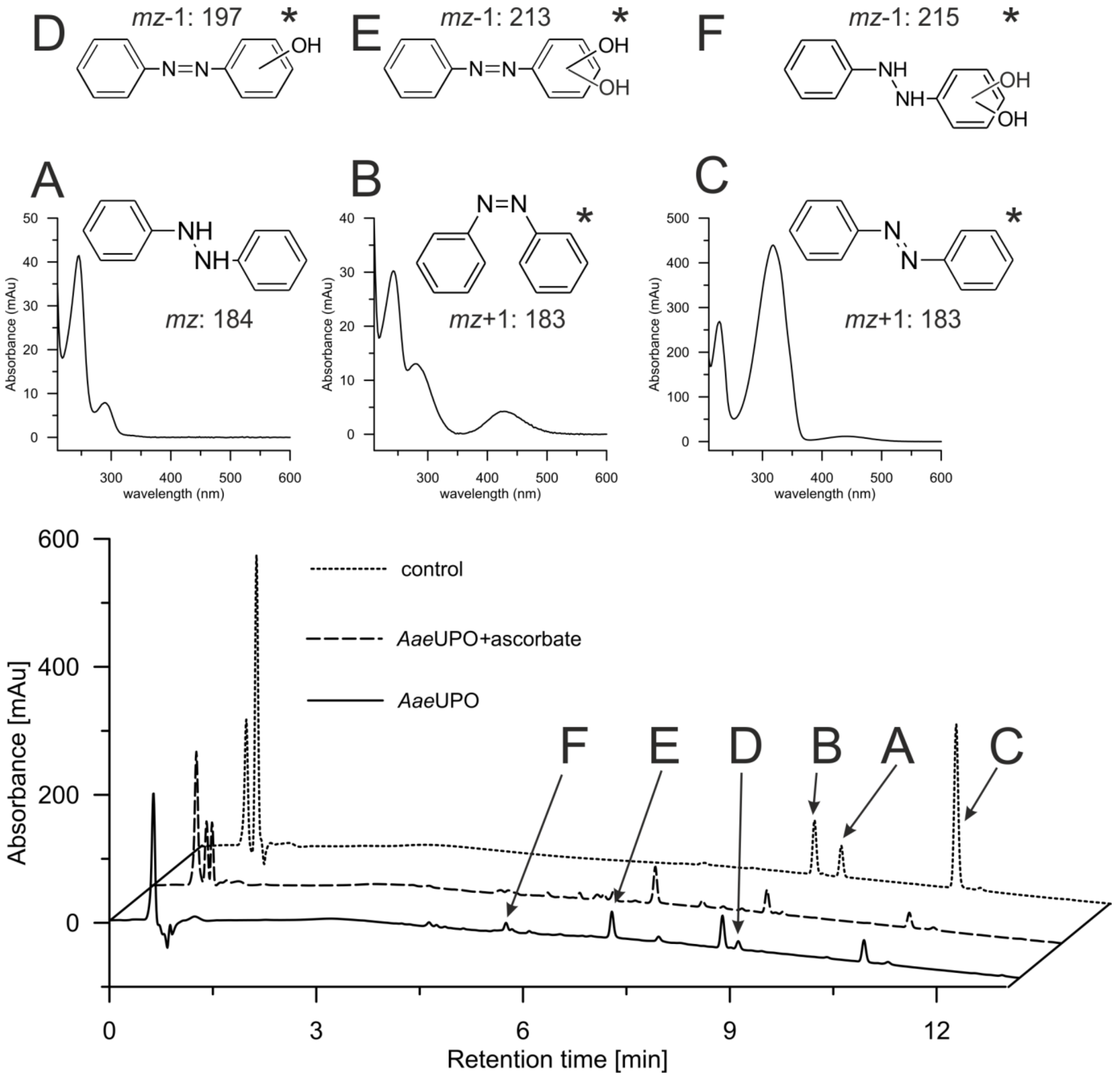

Supplementary figure 23: HPLC-elution profiles (210 nm) of following reaction setup: 1 mM 1,2 diphenylhydrazine (A), 1 mM H_2O_2, 0.45 U_{Valk}/mL *Aae*UPO, 50 mM KPi pH 7, 5% acetonitrile, 4 mM (ascorbate if added). The total reaction volume was 500 µL; addition of H_2O_2 started the reaction. Reaction mixture was analyzed with **Method 3** after 30 min.

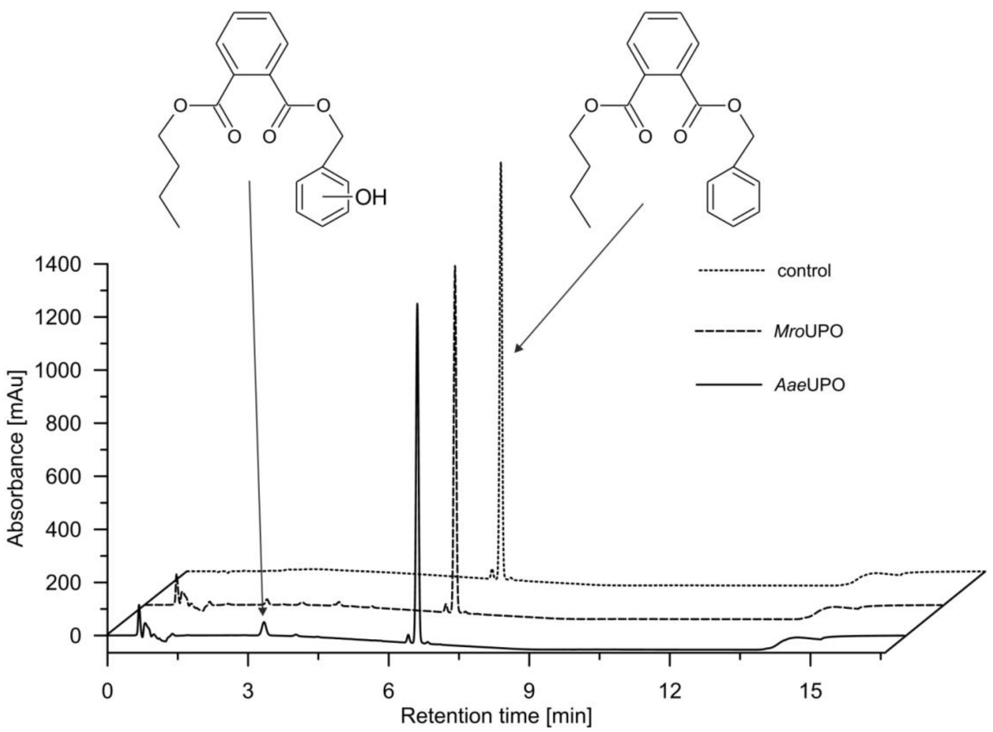

Supplementary figure 24: HPLC-elution profiles (210 nm) of following reaction setup: 0.5 mM butyl benzyl phthalate, 1 mM H_2O_2, 1 U_{Valk}/mL *Aae*UPO/*Mro*UPO, 40 mM KP_i pH 7, 25% acetonitrile. The total reaction volume was 500 µL; addition of H_2O_2 started the reaction. Reaction mixture was diluted with acetonitrile (75%) 1:1 (vol/vol) prior to injection into the HPLC system. Reaction mixture was analyzed with **Method 5** after 30 min.

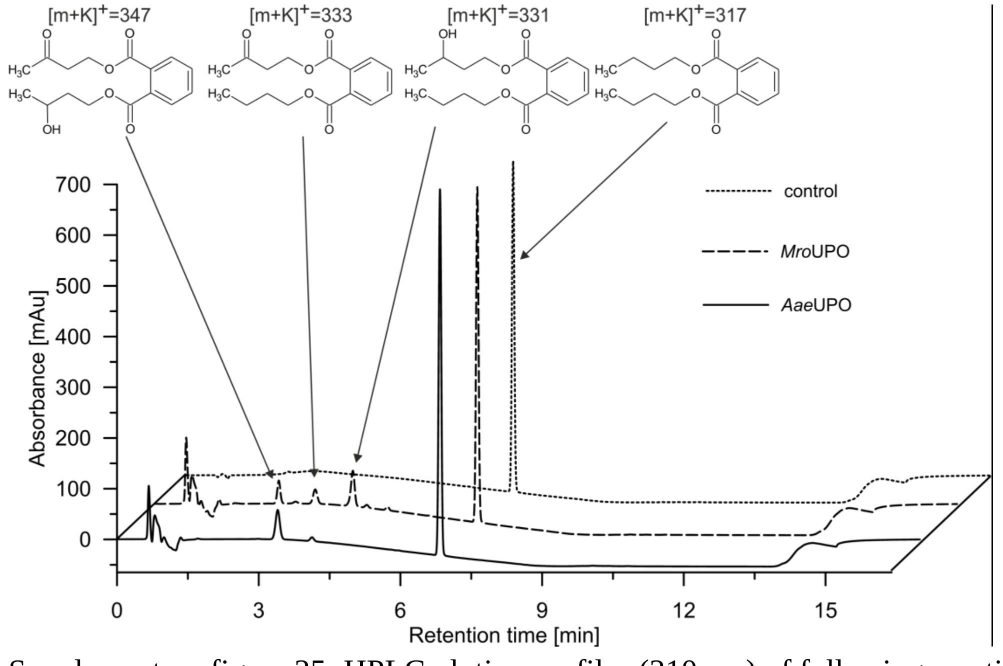

Supplementary figure 25: HPLC-elution profiles (210 nm) of following reaction setup: 0.5 mM di-*n*-butyl phthalate, 1 mM H_2O_2, 1 U_{Valk}/mL *Aae*UPO/*Mro*UPO, 40 mM KP_i pH 7, 25% acetonitrile. Reaction mixture was diluted with acetonitrile (75%) 1:1 (vol/vol) prior to

injection. The total reaction volume was 500 µL; addition of H_2O_2 started the reaction. Reaction mixture was analyzed with **Method 5** after 30 min.

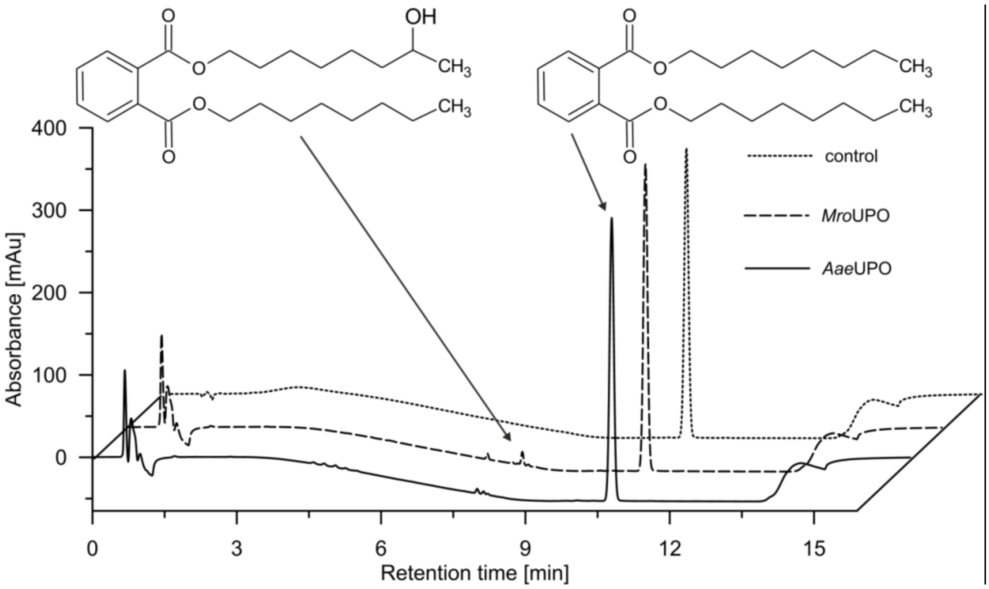

Supplementary figure 26: HPLC-elution profiles (210 nm) of following reaction setup: 0.5 mM di-*n*-octyl phthalate, 1 mM H_2O_2, 1 U_{Valk}/mL *Aae*UPO/*Mro*UPO, 40 mM KP_i pH 7, 25% acetonitrile. Reaction mixture was diluted with acetonitrile (75%) 1:1 prior to injection. The total reaction volume was 500 µL; addition of H_2O_2 started the reaction. Reaction mixture was analyzed with **Method 5** after 30 min.

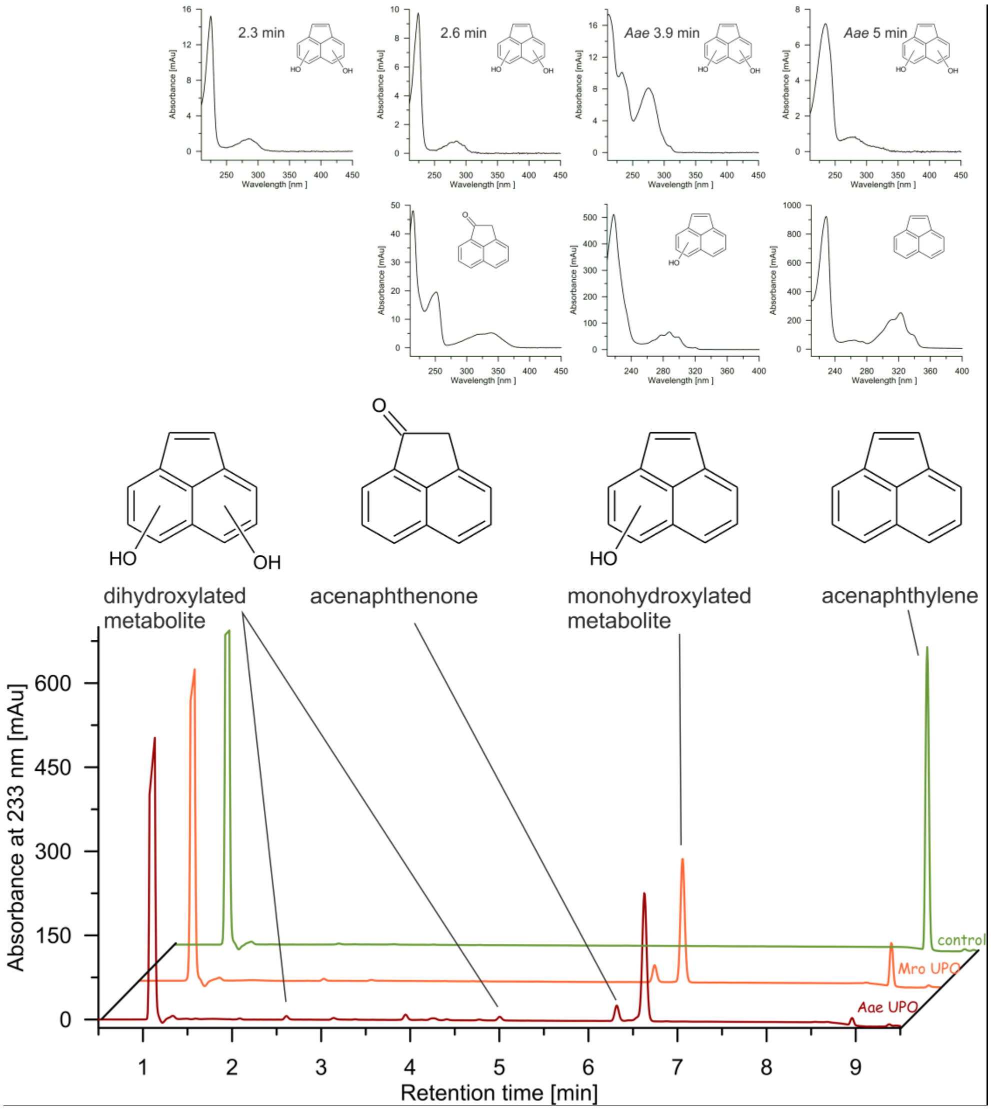

Supplementary figure: HPLC-elution profiles (233 nm) of following reaction setup: 0.5 mM acenaphthylene, 0.5 mM H_2O_2, 1 U_{Valk}/mL *Aae*UPO/*Mro*UPO, 50 mM KP_i pH 7, 10% acetonitrile, 2 mM ascorbic acid, addition of H_2O_2 started the reaction. The total reaction volume was 500 µL. Reaction was stopped with 100 µL 13 mM sodium azide and diluted with 400 µL acetonitrile prior to analysis. Reaction mixture was analyzed with **Method 6.**

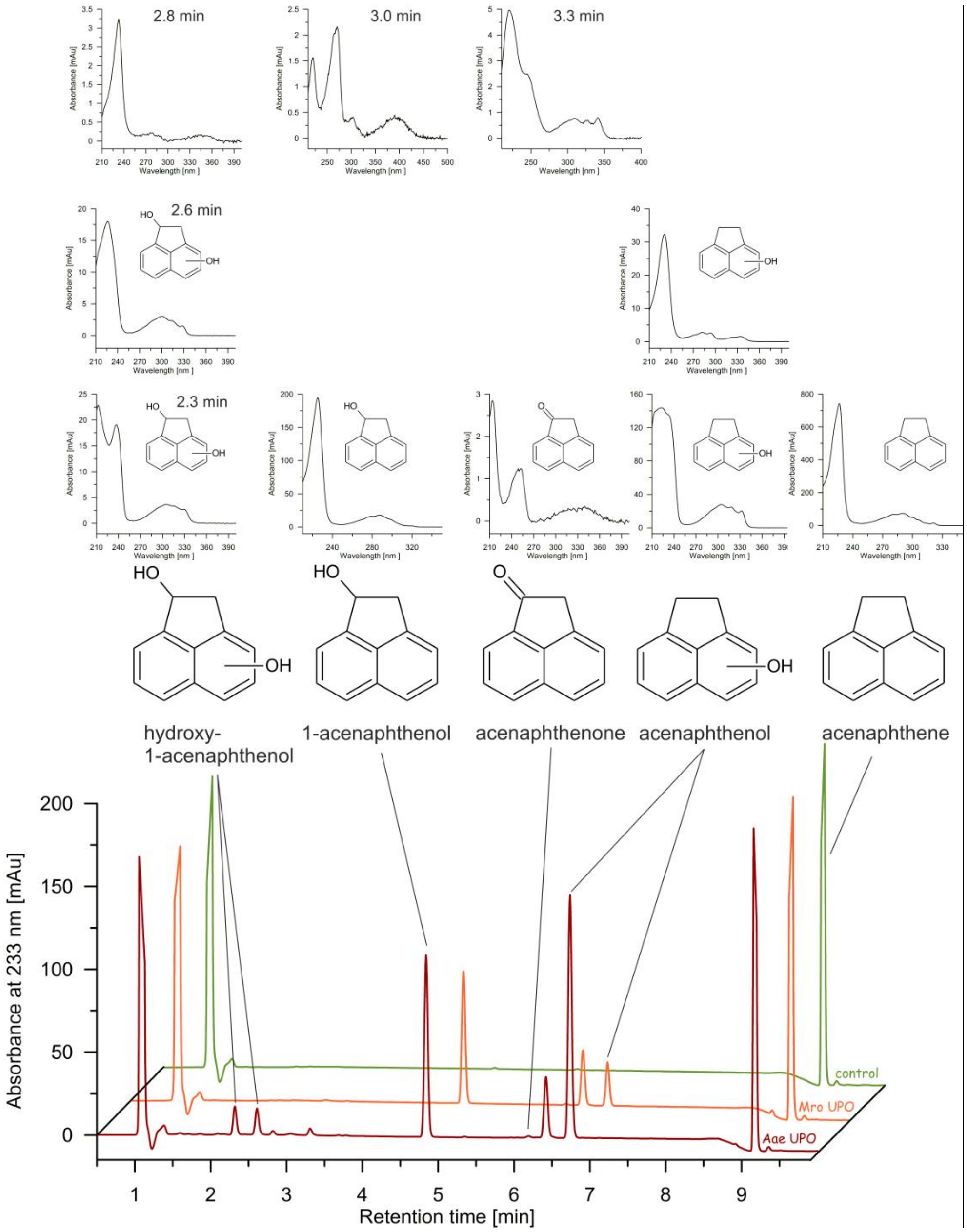

Supplementary figure. 28: HPLC-elution profiles (233 nm) of following reaction setup: 0.5 mM acenaphthene, 0.5 mM H_2O_2, 1 U_{Valk}/mL *Aae*UPO/*Mro*UPO, 50 mM KP_i pH 7, 10% acetonitrile, 2 mM ascorbic acid, addition of H_2O_2 started the reaction. The total reaction volume was 500 µL. Reaction was stopped with 100 µL 13 mM sodium azide and diluted with 400 µL acetonitrile prior to analysis. Reaction mixture was analyzed with **Method 6.**

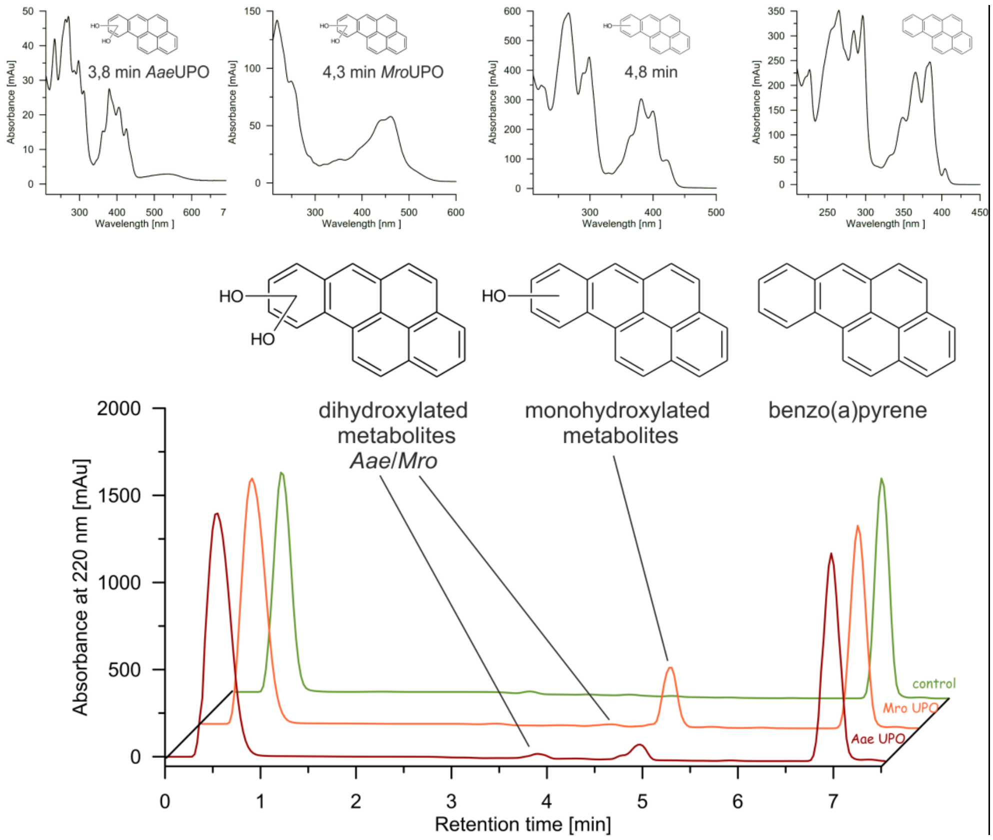

Supplementary figure 29: HPLC-elution profiles (220 nm) of following reaction setup: 0.5 mM benzo[a]pyrene, 1 mM H_2O_2, 1 U_{Valk}/mL *Aae*UPO/*Mro*UPO, 50 mM KP_i pH 7, 30% acetonitrile, 2 mM ascorbic acid; addition of H_2O_2 started the reaction. The total reaction volume was 500 µL. Reaction was stopped with 100 µL 13 mM sodium azide and the mixture diluted with 400 µL acetone prior to analysis. Reaction mixture was analyzed with **Method 10.**

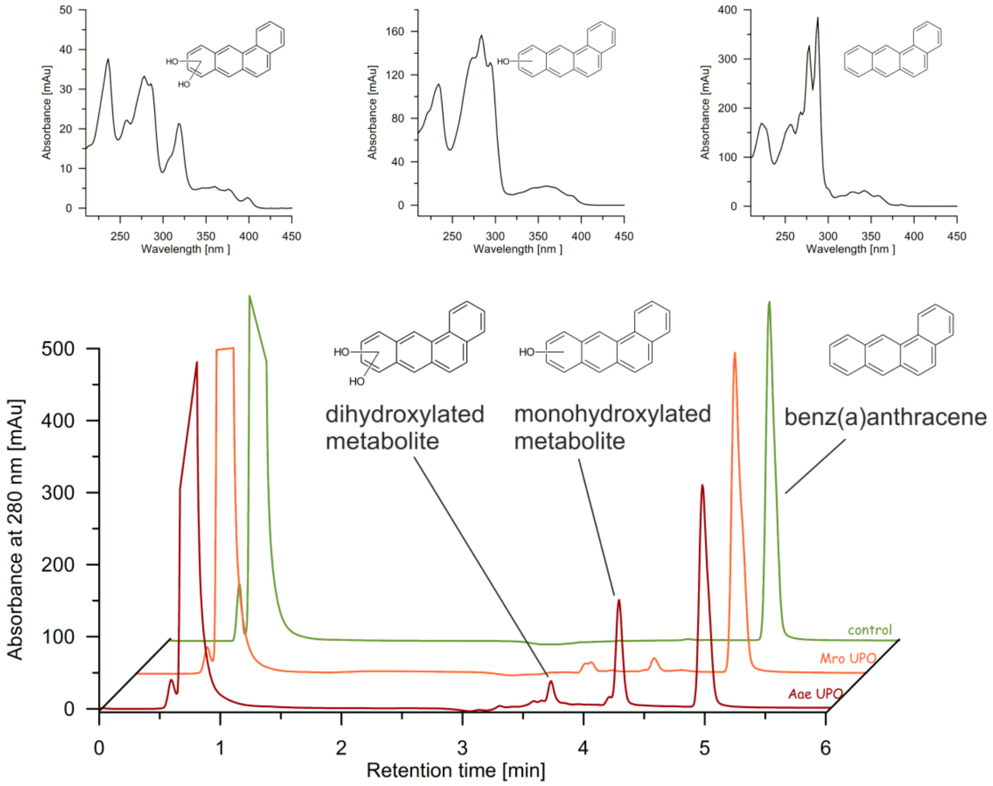

Supplementary figure. 30: HPLC-elution profiles (280 nm) of following reaction setup: 0.5 mM benz[a]anthracene, 0.5 mM H_2O_2, 1 U_{Valk}/mL *Aae*UPO/*Mro*UPO, 50 mM KP_i pH 7, 20% acetonitrile, 2 mM ascorbic acid; addition of H_2O_2 was realized with a syringe pump over 2 h and started the reaction. The total reaction volume was 500 µL. Reaction was stopped with 100 µL 13 mM sodium azide and the mixture diluted with 400 µL acetone prior to analysis. Reaction mixture was analyzed with **Method 7.**

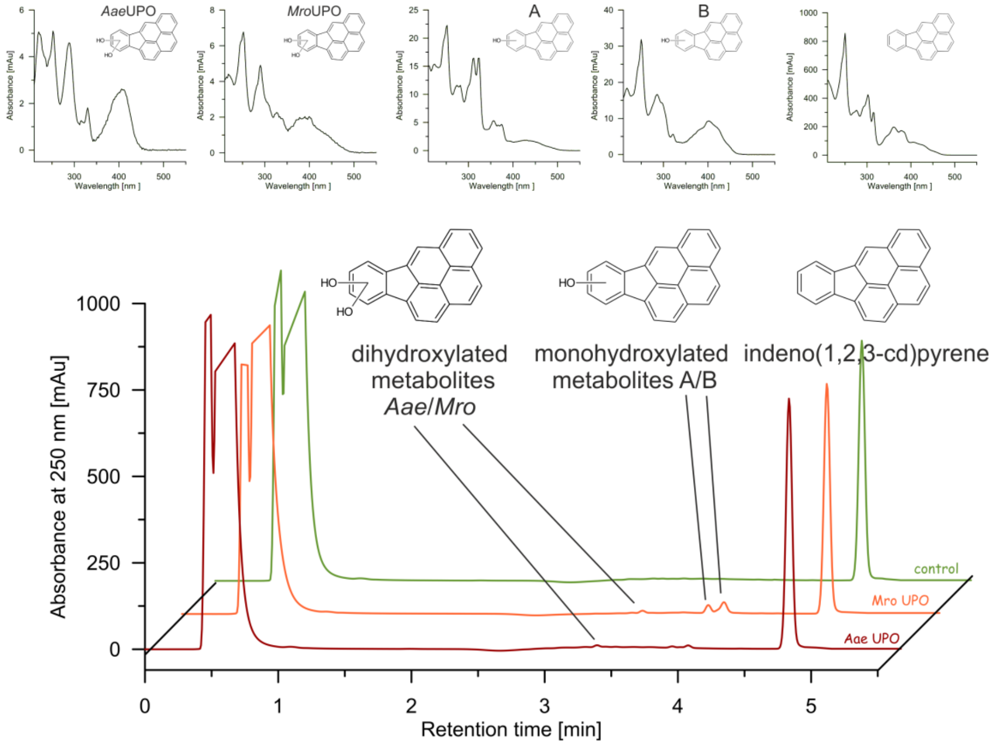

Supplementary figure 31: HPLC-elution profiles (250 nm) of following reaction setup: 0.5 mM indeno[1,2,3-cd]pyrene, 0.1 mM H_2O_2, 1 U_{Valk}/mL *Aae*UPO/*Mro*UPO, 50 mM KP_i pH 7, 20% acetonitrile, 2 mM ascorbic acid; addition of H_2O_2 was realized with a syringe pump (2 h) and started the reaction. The total reaction volume was 500 µL. Reaction was stopped with 100 µL 13 mM sodium azide and the mixture diluted with 400 µL acetone prior to analysis. Reaction mixture was analyzed with **Method 8.**

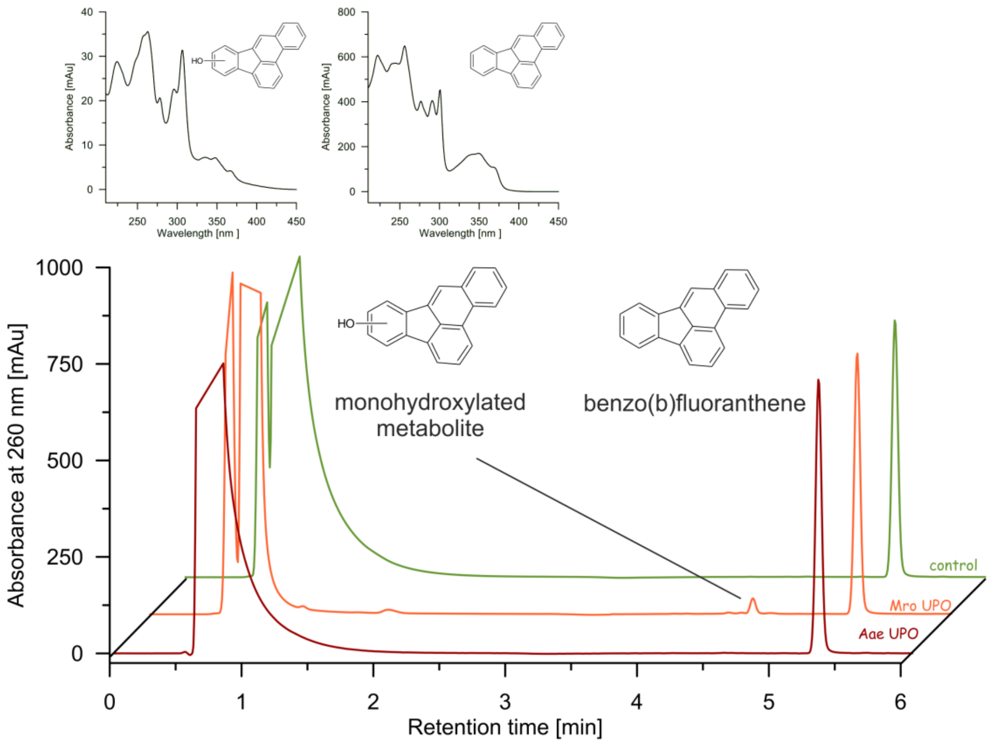

Supplementary figure 32: HPLC-elution profiles (260 nm) of following reaction setup: 0.1 mM benzo[b]fluoranthene, 0.2 mM H_2O_2, 1 U_{Valk}/mL *Aae*UPO/*Mro*UPO, 50 mM KP_i pH 7, 20% acetonitrile, 2 mM ascorbic acid; addition of H_2O_2 was realized with a syringe pump over 2 h and started the reaction. The total reaction volume was 500 µL. Reaction was stopped with 100 µL 13 mM sodium azide and the mixture diluted with 400 µL acetone prior to analysis. Reaction mixture was analyzed with **Method 7.**

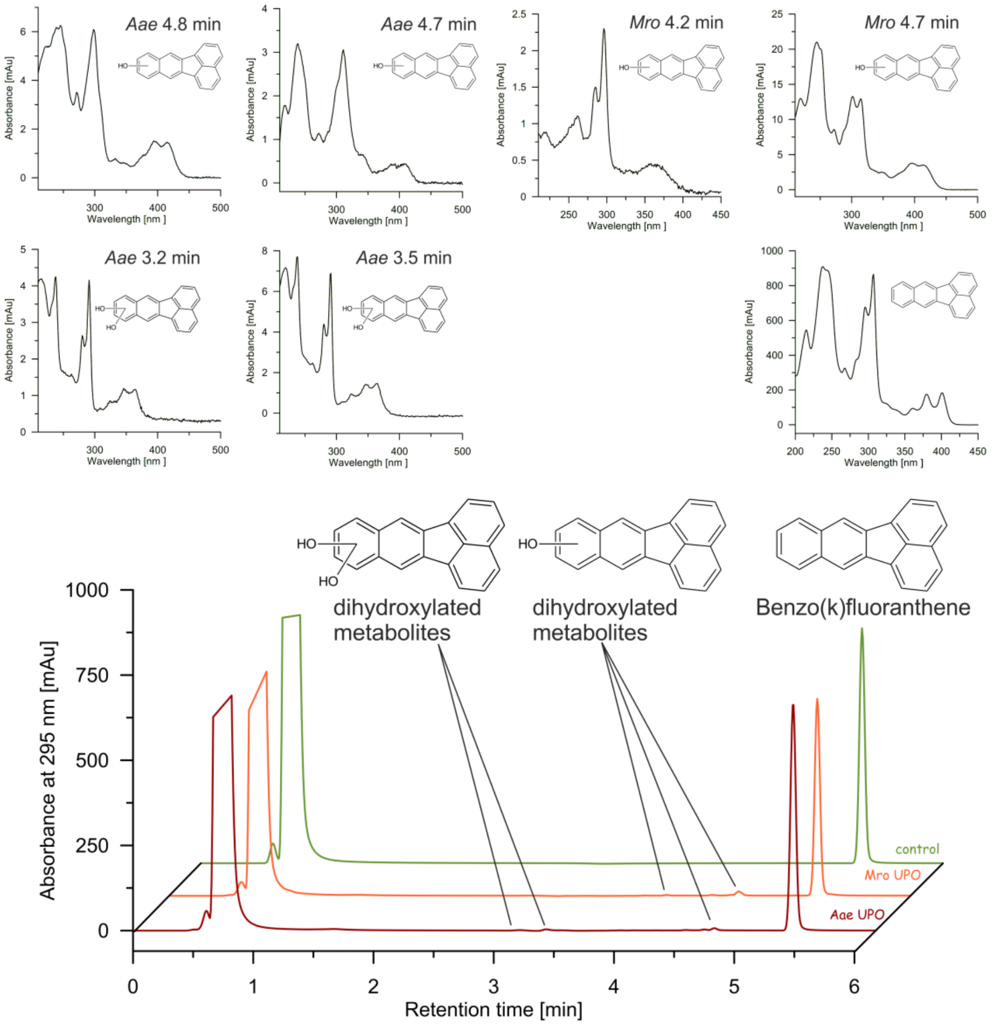

Supplementary figure 33: HPLC-elution profiles (295 nm) of following reaction setup: 0.1 mM benzo[k]fluoranthene, 0.2 mM H_2O_2, 1 U_{Valk}/mL *Aae*UPO/*Mro*UPO, 50 mM KP_i pH 7, 20% acetonitrile, 2 mM ascorbic acid, addition of H_2O_2 was realized with a syringe pump (2 h) and started the reaction. The total reaction volume was 500 µL. Reaction was stopped with 100 µL 13 mM sodium azide and the mixture diluted with 400 µL acetone prior to analysis. Reaction mixture was analyzed with **Method 7.**

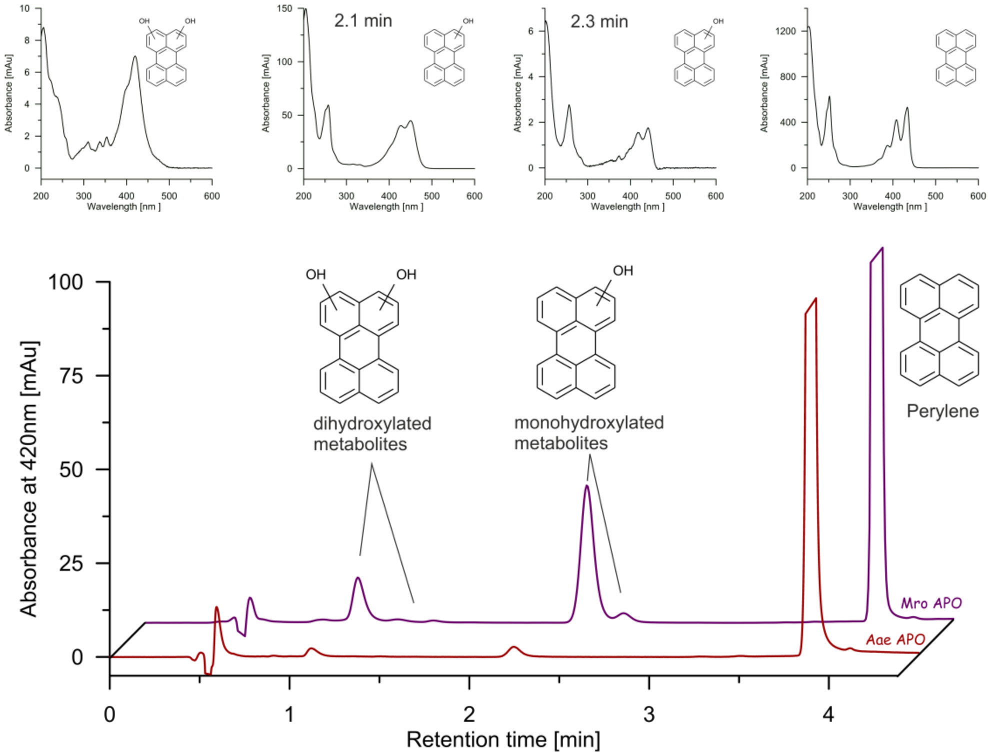

Supplementary figure 34: HPLC-elution profiles (420 nm) of following reaction setup: 0.1 mM perylene, 0.5 mM H_2O_2, 1 U_{Valk}/mL *Aae*UPO/*Mro*UPO, 50 mM KP_i pH 7, 20% acetonitrile, 2 mM ascorbic acid; supply with H_2O_2 was realized with a syringe pump (2 h) and started the reaction. The total reaction volume was 500 µL. Reaction was stopped with 100 µL 13 mM sodium azide and the mixture diluted with 400 µL acetone prior to analysis. Reaction mixture was analyzed with **Method 9.**

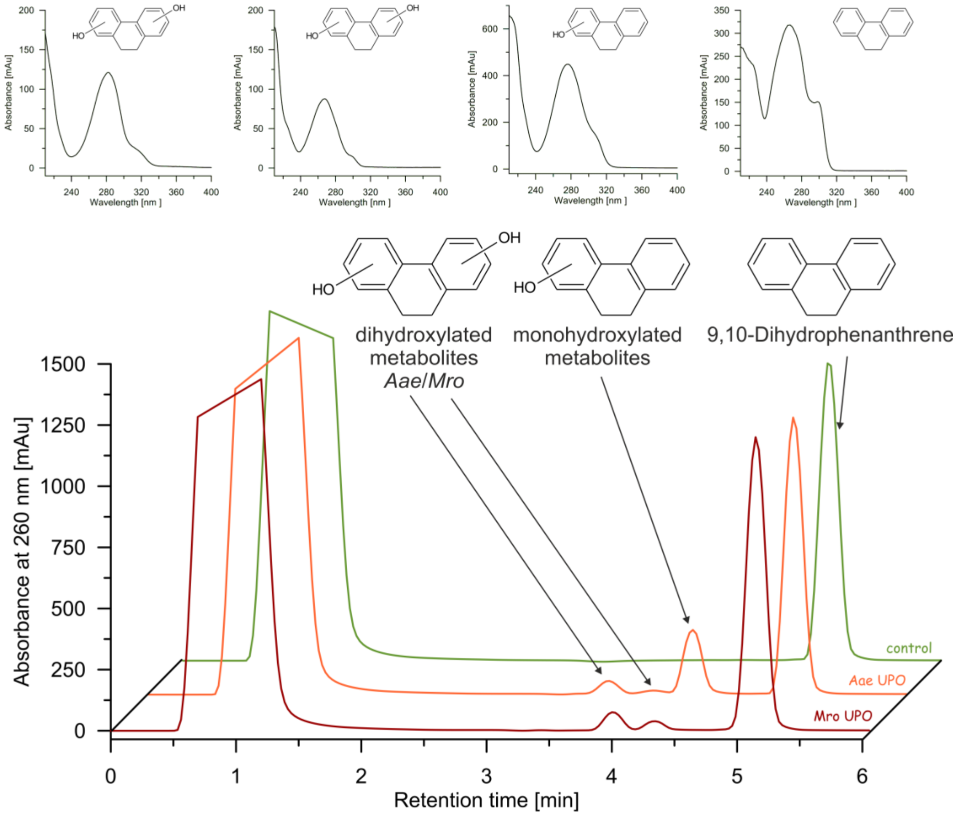

Supplementary figure 35: HPLC-elution profiles (260 nm) of following reaction setup: 1 mM 9,10-dihydrophenanthrene, 1 mM H_2O_2, 1 U_{Valk}/mL *Aae*UPO/*Mro*UPO, 50 mM KP_i pH 7, 30% acetonitrile, 2 mM ascorbic acid; supply with H_2O_2 was realizedusing a syringe pump (2 h) and started the reaction. The total reaction volume was 500 µL. Reaction was stopped with 100 µL 13 mM sodium azide and the mixture diluted with 400 µL acetone prior to analysis. Reaction mixture was analyzed with **Method 10.**

HPLC analysis methods

Reaction products were analyzed by HPLC using an Agilent Series 1200 instrument equipped with a diode array detector (Agilent Technologies Deutschland GmbH, Böblingen, Germany) following the methods listed below.

Method 1
Column: Luna C18(2), 5µm, 100Å, 150 x 2 mm (Phenomenex, Aschaffenburg, Germany)
Eluent A: 0,01% formic acid set to pH 3.6 with ammonia (25%)
Eluent B: acetonitrile
Flow: 0.5 mL/min
Oven temperature: 45°C
Injection volume: 10 µL

Gradient:

time	% B
0	7
1	7
2	15
4	30
12	35
13	100

Method 2
Column: Luna C18(2), 5µm, 100 Å, 150 x 2 mm (Phenomenex, Aschaffenburg, Germany)
Eluent A: 0,01% formic acid set to pH 3.6 with ammonia (25%)
Eluent B: acetonitrile
Flow: 0.5 mL/min
Oven temperature: 45°C
Injection volume: 10 µl

Gradient:

time	% B
0	5
1	5
7	50
15	60
15	100

Method 3
Column: Kinetex PFP, 2.6 µm, 100 Å, 150 x 2.1 mm (Phenomenex, Aschaffenburg, Germany)
Eluent A: 0,01% formic acid set to pH 3.6 with ammonia (25%)
Eluent B: acetonitrile
Flow: 0.6 mL/min
Oven temperature: 40°C
Injection volume: 10 µl

time	% B
0	5

0.5	5	Gradient:
11	95	

Method 4

Column: Kinetex PFP, 2.6 µm, 100 Å, 150 x 2.1 mm (Phenomenex, Aschaffenburg, Germany)

Eluent A: 0,01% formic acid set to pH 3.6 with ammonia (25%)

Eluent B: acetonitrile

Flow: 0.6 mL/min

Oven temperature: 40°C

Injection volume: 5 µl

Gradient:

time	% B
0	5
0.5	5
11	95

Method 5

Column: Luna C18(2), 5µm, 100 Å, 150 x 2 mm (Phenomenex, Aschaffenburg, Germany)

Eluent A: 0,01% formic acid set to pH 3.6 with ammonia (25%)

Eluent B: acetonitrile

Flow: 0.5 mL/min

Oven temperature: 40°C

Injection volume: 10 µl

Gradient:

time	% B
0	50
0.5	50
6	95

Method 6

Column: Kinetex C18, 5µm, 100 Å, 250 x 4.6 mm (Phenomenex, Aschaffenburg, Germany)

Eluent A: 0,01% formic acid set to pH 3.6 with ammonia (25%)

Eluent B: acetonitrile

Flow: 0.5 mL/min

Oven temperature: 45°C

Injection volume: 10 µl

time	% B
0	30

		Gradient:
0.5	30	
7	60	
7.5	95	

Method 7
Column: Kinetex PFP, 2.6µm, 100 Å, 100 x 2.1 mm (Phenomenex, Aschaffenburg, Germany)
Eluent A: 0,01% formic acid set to pH 3.6 with ammonia (25%)
Eluent B: acetonitrile
Flow: 0.5 mL/min
Oven temperature: 45°C
Injection volume: 5 µl

Gradient:

time	% B
0	25
0.5	25
1	70
6	78
7	95

Method 8
Column: Kinetex PFP, 2.6µm, 100 Å, 100 x 2.1 mm (Phenomenex, Aschaffenburg, Germany)
Eluent A: 0,01% formic acid set to pH 3.6 with ammonia (25%)
Eluent B: acetonitrile
Flow: 0.5 mL/min
Oven temperature: 45°C
Injection volume: 5 µl

Gradient:

time	% B
0	25
0.5	25
1	70.5
3	71.5
4	95

Method 9
Column: Kinetex PFP, 2.6µm, 100 Å, 150 x 2.1 mm (Phenomenex, Aschaffenburg, Germany)
Eluent A: 0,01% formic acid set to pH 3.6 with ammonia (25%)

Eluent B: acetonitrile
Flow: 0.5 mL/min
Oven temperature: 45°C
Injection volume: 10 µl

Gradient:

time	% B
0	50
1	50
2.5	95

Method 10
Column: Synergi polar, 2.5µm, 100 Å, 120 x 2 mm (Phenomenex, Aschaffenburg, Germany)
Eluent A: 0,01% formic acid set to pH 3.6 with ammonia (25%)
Eluent B: acetonitrile
Flow: 0.4 mL/min
Oven temperature: 30°C
Injection volume: 20 µl

Gradient:

time	% B
0	25
0.5	25
1	70
6	78
7	95

Method 11
Column: Kinetex PFP, 2.6µm, 100 Å, 100 x 2.1 mm (Phenomenex, Aschaffenburg, Germany)
Eluent A: 0,01% formic acid set to pH 3.6 with ammonia (25%)
Eluent B: acetonitrile
Flow: 0.4 mL/min
Oven temperature:45°C
Injection volume: 10 µl

Gradient:

time	% B
0	8
5	8
9	95

<u>Mass analysis</u>

An electrospray ionization mass spectrometer (6310 IonTrap, Agilent Technologies Deutschland GmbH) was used to determine mass-to-charge ratios of substrates tested and metabolites formed upon reaction of UPOs.
